# IRF-7 Mediates Type I IFN Responses in Endotoxin-Challenged Mice

**DOI:** 10.3389/fimmu.2020.00640

**Published:** 2020-04-16

**Authors:** Wei-Xiang Sin, Joe Poh-Sheng Yeong, Thomas Jun Feng Lim, I-Hsin Su, John E. Connolly, Keh-Chuang Chin

**Affiliations:** ^1^Institute of Molecular and Cell Biology, Agency for Science, Technology and Research, Singapore, Singapore; ^2^Division of Pathology, Singapore General Hospital, Singapore, Singapore; ^3^School of Biological Sciences, College of Science, Nanyang Technological University, Singapore, Singapore; ^4^Institute of Biomedical Studies, Baylor University, Waco, TX, United States; ^5^Department of Physiology, NUS Yong Loo Lin School of Medicine, National University of Singapore, Singapore, Singapore

**Keywords:** IRF-7, TLR4, IFN-β, IL-1β, macrophage, dendritic cell

## Abstract

IRF-7 mediates robust production of type I IFN via MyD88 of the TLR9 pathway in plasmacytoid dendritic cells (pDCs). Previous *in vitro* studies using bone marrow-derived dendritic cells lacking either *Irf7* or *Irf3* have demonstrated that only IRF-3 is required for IFN-β production in the TLR4 pathway. Here, we show that IRF-7 is essential for both type I IFN induction and IL-1β responses via TLR4 in mice. Mice lacking *Irf7* were defective in production of both IFN-β and IL-1β, an IFN-β-induced pro-inflammatory cytokine, after LPS challenge. IFN-β production in response to LPS was impaired in IRF-7-deficient macrophages, but not dendritic cells. Unlike pDCs, IRF-7 is activated by the TRIF-, but not MyD88-, dependent pathway via TBK-1 in macrophages after LPS stimulation. Like pDCs, resting macrophages constitutively expressed IRF-7 protein. This basal IRF-7 protein was completely abolished in either *Ifnar1*^−/−^ or *Stat1*^−/−^ macrophages, which corresponded with the loss of LPS-stimulated IFN-β induction in these macrophages. These findings demonstrate that macrophage IRF-7 is critical for LPS-induced type I IFN responses, which in turn facilitate IL-1β production in mice.

## Introduction

Sepsis is one of the leading causes of morbidity and mortality in hospital intensive care units worldwide (1). It is a systemic inflammatory response to severe microbial infections that is characterized by the excessive production of pro-inflammatory cytokines. Interleukin-1β (IL-1β) is one of the more studied pro-inflammatory cytokines, and is produced in response to the endotoxins from the outer membrane of the cell wall of Gram-negative bacteria. Excessive or inappropriate expression of IL-1β also occurs with tissue damage and various diseases, including autoimmune diseases, metabolic syndromes, and cryopyrin-associated periodic syndromes (2). The generation of active IL-1β from precursor IL-1β requires the assembly of multiple cytosolic proteins into a complex known as the inflammasome, which acts as a signaling platform to promote the activation of caspase-1 that cleaves pro-IL-1β into active mature IL-1β (3–5). The most extensively studied inflammasome complex to date is the NOD-like receptor pyrin domain-containing protein 3 (NLRP3) inflammasome, which can be activated both in a canonical and in a non-canonical manner (6).

In the non-canonical NLRP3 inflammasome pathway, IL-1β induction in mice and humans after Gram-negative bacterial infections required interferon (IFN)-inducible caspase-11 in mice, or caspase-4/5 in humans (7–9). This response is mediated by Toll-like receptor 4 (TLR4), a receptor that recognizes the lipopolysaccharide (LPS) component of Gram-negative bacteria. TLR4 is the only member in the TLR family that transduces signals via two distinct intracellular pathways, namely the myeloid differentiation primary response protein 88 (MyD88)- and Toll/interleukin-1 receptor (TIR) domain-containing adapter protein inducing interferon-β (TRIF)-dependent pathways. As in the canonical NLRP3 inflammasome pathway, the initial binding of LPS to TLR4 at the plasma membrane recruits the adaptor proteins MyD88 and MyD88 adapter-like (Mal), also termed TIRAP, which induce the activation of nuclear factor kappa-light-chain-enhancer of activated B cells (NF-κB) and mitogen-activated protein kinases (MAPKs), and thus promotes the expression of pro-inflammatory cytokine genes, including pro-IL-1β (10). Subsequently, endocytosis of TLR4 into endosomal compartments initiates a second signaling cascade mediated by the adaptor proteins TRIF and TRAM. This endosomal TRIF-TRAM axis activates TANK-binding kinase 1 (TBK-1) and I-κB kinase ε (IKK-ε), consequently inducing the phosphorylation and nuclear translocation of transcription factor interferon regulatory factor 3 (IRF-3) to promote the expression of type I IFN genes (11–18). In mice, this TRIF-dependent type I IFN production and signaling is required for non-canonical NLRP3 inflammasome activation via transcriptional induction of *Casp11*. Cytoplasmic LPS from Gram-negative bacteria binds to and activates caspase-11, thereby resulting in IL-1β processing and release in a NLRP3-dependent and caspase-1-dependent manner (9, 19–21). In agreement with this model, mice lacking TRIF or IFN-α/β receptor (IFNAR) exhibited defective IL-1β production in response to Gram-negative bacterial infection. In addition, neutralization of IFN-β decreased serum IL-1β levels after LPS challenge. These results support the notion that TRIF is required for LPS-induced IL-1β expression via type I IFN and IFN-induced caspase-11 *in vivo* (9, 22).

IRF-3 and IRF-7 are key transcriptional factors for type I IFN expression. Whilst IRF-3 is constitutively expressed in all cell types, IRF-7 is constitutively expressed only in plasmacytoid dendritic cells (pDCs), while in most of the other cell types it is expressed only after viral infection (23, 24). It was previously demonstrated that TRIF is able to interact with and activate both IRF-7 and IRF-3 (25, 26), which suggests that type I IFN induction in the TLR4-TRIF pathway may be mediated by both IRF-7 and IRF-3. However, it was reported that bone marrow-derived dendritic cells (BMDCs) from *Irf7*-deficient mice exhibited normal IFN-β induction by TLR4 stimulation, whereas IFN-β production was severely impaired in *Irf* 3-deficient BMDCs (24). As macrophages and dendritic cells (DCs) originate from the same myeloid progenitors, and both cell types sense LPS via TLR4 to activate cytokine production via common MyD88 and TRIF pathways, the general consensus is that TLR4-induced IFN-β expression in macrophages is mediated by IRF-3 alone, as is the case in DCs (27). However, several reports have demonstrated that macrophages and DCs can display distinct effector functions in innate immune responses. While both MyD88- and TRIF-dependent pathways are required for sustained activation of NF-κB and pro-inflammatory cytokine production following LPS recognition by TLR4 in bone marrow-derived macrophages (BMDMs) (28), BMDC production of pro-inflammatory cytokines is dependent on MyD88, but independent of TRIF (29, 30). Furthermore, it has been shown that CD11b acts as a cell-type specific regulator to positively promote TLR4 signaling in DCs, but not in macrophages (31).

In this report, we used an established mouse model of LPS-induced acute septic shock to evaluate the role of IRF-7 in the activation of IL-1β and expression of type I IFN responses *in vivo*. According to our studies, mice lacking either IRF-7 or IRF-3 failed to produce IL-1β, and this correlated strongly with a severe defect in IFN-α/β production. From these findings, we conclude that IRF-7 and IRF-3 co-operate in the promotion of IFN-β and IL-1β production *in vivo*. Our studies using *in vitro* cultured bone marrow-derived macrophages and DCs allowed us to identify IRF-7 as a cell type-specific regulator in macrophages, but not in DCs. IRF-7, together with IRF-3, promotes type I IFN production in LPS-stimulated macrophages. Similar to pDCs, IRF-7 is constitutively expressed in resting macrophages, but not in DCs. This expression is dependent on basal IFN-β signaling that is present in macrophages, but not in DCs. In conclusion, our current study shows that IRF-7 is functionally important for the activation of type I IFN production in the TLR4 signaling pathway in macrophages, contrary to the previous conclusion that IRF-7 is completely dispensable in DCs.

## Materials and Methods

### Mice

All mice were derived from a C57BL/6 genetic background. MyD88-deficient (MyD88^−/−^) mice were from OrientalBioService, Inc. (Kyoto, Japan). TRIF-deficient (Ticam1^Lps2^/J) mice were from The Jackson Laboratory (Bar Harbor, Maine, USA). IFNAR1-deficient (Ifnar1^tm1Agt^/Mmjax) mice were from Mutant Mouse Regional Resource Centers (MMRRC), National Institutes of Health (NIH) (Bethesda, Maryland, USA). STAT1-deficient (Stat1^tm1Rds^) mice were from Taconic Biosciences, Inc. (Hudson, NY, USA). IRF-3-deficient (IRF-3^−/−^) and IRF-7-deficient (IRF-7^−/−^) mice were from RIKEN BioResource Center (Ibaraki, Japan). IRF-3-IRF-7 double knockout mice were generated in-house by intercrossing IRF-3^−/−^ and IRF-7^−/−^ mice. Homozygous IRF-3^−/−^-IRF-7^−/−^ mice were generated by intercrossing heterozygous IRF-3^+/−^-IRF-7^+/−^ F1 mice, and were verified by genotyping tail biopsies. Bone marrow cells were obtained from STAT3 conditional knockout (MxCre-STAT3f/f) mice and control mice lacking the Mx-Cre transgene (STAT3f/f) (kind gift of Chien-Kuo Lee, National Taiwan University College of Medicine, Taiwan, Republic of China). All mice were bred and maintained at the A^*^STAR Biological Resource Center under specific pathogen-free conditions. All animal experimental procedures were conducted within the parameters of our Institutional Animal Care and Use Committee (IACUC)-approved protocol, in compliance with the National Advisory Committee for Laboratory Animal Research (NACLAR) Guidelines.

### Preparation of Murine Bone Marrow Cells

Mice were euthanized using carbon dioxide followed by cervical dislocation to ensure death. After euthanasia, femurs and tibias were dissected from each mouse using scissors and forceps, and the bones were placed into a petri dish containing DMEM complete medium. Both epiphyses were removed from each bone using scissors and forceps, and bone marrow cells were flushed into a 50-ml polypropylene tube using a 25-G needle and a 10-ml syringe containing DMEM complete medium. After centrifugation at 500 g for 10 min, the cell pellet was resuspended in 3 ml Red Blood Cell (RBC) lysis buffer for 3 min at room temperature. RBC lysis was stopped by adding 10 ml DMEM complete medium. After centrifugation at 500 g for 10 min, the cell pellet was resuspended in freezing medium (FBS + 10% DMSO). Bone marrow cells were aliquoted into cryogenic vials, and then frozen in liquid nitrogen.

### Differentiation of Murine Bone Marrow-Derived Macrophages

Frozen bone marrow cells were thawed in a 37°C water bath and transferred to a 15-ml polypropylene tube containing 10 ml DMEM complete medium. After centrifugation at 500 g for 10 min, the cell pellet was resuspended in BMDM differentiation medium (50% DMEM + 4,500 mg/L glucose + 110 mg/L sodium pyruvate supplemented with 20% HyClone defined FBS and 30% L929 cell-conditioned medium, and 100 U/ml penicillin + 100 μg/ml streptomycin). Bone marrow cells were counted using trypan blue solution (Sigma-Aldrich, St. Louis, MO, USA) and a hemocytometer. For analysis of RNA and culture supernatants, 0.5 × 10^6^ BM cells were cultured in each well of a 6-well plate containing 1.5 ml BMDM differentiation medium. For protein experiments, 1.5 × 10^6^ BM cells were cultured in 60-mm dishes that contained 2.5 ml BMDM differentiation medium. For analysis of nuclear extracts, 6.0–7.0 × 10^6^ BM cells were cultured in 100-mm dishes that contained 10.0 ml BMDM differentiation medium. For ChIP experiments, 20.0 × 10^6^ BM cells were cultured in 150-mm dishes that contained 20.0 ml BMDM differentiation medium. On Day 3, an equivalent volume of fresh BMDM differentiation medium was added to the culture. On Day 5 and Day 6, the BMDM differentiation medium was aspirated and fresh BMDM differentiation medium was added to the adherent cells. On Day 7, BMDMs were used for experiments, and samples were harvested for downstream analysis. Differentiation of bone marrow progenitors into BMDMs was confirmed by flow cytometric analysis of F4/80 and CD11b surface marker expression.

### Differentiation of Murine Bone Marrow-Derived Dendritic Cells

Frozen bone marrow cells were thawed in a 37°C water bath and transferred to a 15-ml polypropylene tube containing 10 ml RPMI complete medium. After centrifugation at 500 g for 10 min, the cell pellet was resuspended in BMDC differentiation medium (90% RPMI 1640 + 10 mM HEPES supplemented with 10% HyClone defined FBS and 20 ng/ml GM-CSF, and 100 U/ml penicillin + 100 μg/ml streptomycin). Bone marrow cells were counted using trypan blue solution (Sigma-Aldrich, St. Louis, MO, USA) and a hemocytometer. 1.5 × 10^6^ BM cells were cultured in each well of a 24-well plate containing 1.0 ml BMDC differentiation medium. On Day 2, an equivalent volume of fresh BMDC differentiation medium was added to the culture. On Day 4, 1.0 ml BMDC differentiation medium was aspirated and 1.0 ml fresh BMDC differentiation medium was added to the culture. On Day 5, the non-adherent cells were collected and re-plated in suspension culture plates for experiments. For analysis of RNA and culture supernatants, 0.5 × 10^6^ BMDCs were cultured in each well of a 24-well suspension culture plate containing 1.5 ml BMDC differentiation medium. For protein experiments, 1.5 × 10^6^ BMDCs were cultured in each well of a 6-well suspension culture plate containing 2.5 ml BMDC differentiation medium. On Day 6, 1.0 ml BMDC differentiation medium was aspirated and 1.0 ml fresh BMDC differentiation medium was added to the culture. On Day 7, BMDCs were used for experiments, and samples were harvested for downstream analysis. Differentiation of bone marrow progenitors into BMDCs was confirmed by flow cytometric analysis of MHCII and CD11c surface marker expression.

### Gene Expression Analysis by Real-Time Quantitative-PCR (qRT-PCR)

Total RNA was harvested using TRIzol Reagent (Ambion, Life Technologies, Carlsbad, California, USA) and isolated by acid guanidinium thiocyanate-phenol-chloroform extraction, followed by purification using the PureLink RNA Mini Kit (Ambion, Life Technologies, Carlsbad, California, USA) according to the manufacturer's instructions. First-strand cDNA was synthesized from 1 μg total RNA per sample by mRNA-specific reverse transcription using Oligo(dT)12-18 Primer and SuperScript III Reverse Transcriptase (Invitrogen, Life Technologies, Carlsbad, California, USA) according to the manufacturer's instructions. The cDNA was used as a template for amplification in qRT-PCR in duplicate. qRT-PCR analysis was performed by SYBR Green (Kapa Biosystems, Inc., Boston, MA, USA) detection using the ABI 7900HT Fast Real-Time PCR System (Applied Biosystems, Life Technologies, Foster City, CA, USA). qRT-PCR primers for gene expression analysis are shown below.

m-Gapdh Forward → ATCTTCTTGTGCAGTGCCAGCCTCGTCCC
m-Gapdh Reverse → TTGACTGTGCCGTTGAATTTGCCGTGAGTG
m-Ifnb1 Forward → CCCTATGGAGATGACGGAGA
m-Ifnb1 Reverse → TCCCACGTCAATCTTTCCTC
m-Irf7 Forward → GCATTTCGGTCGTAGGGATCTGGATGAAGA
m-Irf7 Reverse → CGTACACCTTATGCGGATCAACTGGA

### Protein Expression Analysis by Western Blotting

Total cell lysates were harvested by lysing cells in Radio Immunoprecipitation Assay (RIPA) buffer (25 mM Tris-HCl, pH7.6, 150 mM NaCl (sodium chloride), 1% NP-40, 1% SDS (sodium dodecyl sulfate), 1% sodium deoxycholate) with protease and phosphatase inhibitors (cOmplete, Mini, EDTA-free Protease Inhibitor Cocktail Tablets, Roche Diagnostics, Dubai, UAE; Pierce Phosphatase Inhibitor Tablets, Thermo Fisher Scientific Inc., Rockford, IL, USA) for 1 h at 4°C. Whole cell lysates were clarified by centrifugation at 12,000 rpm for 10 min at 4°C. For nuclear and cytoplasmic lysates, cytoplasmic and nuclear protein fractionation was performed using the NE-PER Nuclear and Cytoplasmic Extraction Kit (Life Technologies, Thermo Fisher Scientific Inc., Rockford, IL, USA). Protein concentrations were determined by the Bradford assay using Protein Assay Dye Reagent Concentrate (Bio-Rad Laboratories, Inc., Hercules, CA, USA) and Tecan Infinite M200 Microplate Reader (Tecan Trading AG, Switzerland) according to the manufacturer's instructions. Protein concentrations were normalized, and sample lysates were denatured by addition of Sodium Dodecyl Sulfate (SDS) loading buffer with β-mercaptoethanol and then boiling for 5 min at 95°C. Equal amounts of sample lysates were separated by 9% Sodium Dodecyl Sulfate PolyAcrylamide Gel Electrophoresis (SDS-PAGE) under reducing and denaturing conditions (Amersham, GE Healthcare Bio-Sciences, Sweden), and transferred onto polyvinylidene difluoride (PVDF) membranes (Amersham, GE Healthcare Bio-Sciences, Sweden). Blots were blocked in 5% milk or BSA solution (for phospho-proteins) to prevent non-specific background binding, and probed with specific antibodies in 5% milk or BSA solution (for phospho-proteins) shown below.

Anti-Actin (MAB1501) was from Merck Millipore (Temecula, CA, USA), anti-α Tubulin (B-7) (sc-5286) and anti-IRF-3 (FL-425) (sc-9082) were from Santa Cruz Biotechnology, Inc. (Dallas, Texas, USA), anti-TATA binding protein TBP [1TBP18] (ab818) was from Abcam (Cambridge, MA, USA), anti-Stat1 (pY701) (612132) and anti-Stat1 (N-Terminus) (610115) were from BD Transduction Laboratories (Franklin Lakes, New Jersey, USA), anti-Phospho-IRF-3 (Ser396) (4947) was from Cell Signaling Technology, Inc. (Danvers, MA, USA), anti-IRF-7 (51-3300) was from Invitrogen (Life Technologies, Carlsbad, California, USA), AffiniPure Donkey anti-rabbit HRP (711-035-152), AffiniPure Donkey anti-mouse HRP (715-035-150), and AffiniPure Donkey anti-goat HRP (705-035-147) were from Jackson ImmunoResearch Inc. (West Grove, PA, USA).

### Enzyme-Linked Immunosorbent Assay (ELISA)

Cytokine levels in culture supernatants were measured using VeriKine Mouse Interferon Beta ELISA Kit (PBL Assay Science, Piscataway, NJ, USA) according to the manufacturer's instructions.

### Chromatin Immunoprecipitation (ChIP) Analysis

DNA and proteins in cells were cross-linked using 1% formaldehyde for 10 min at room temperature and quenched using 200 mM glycine for 1 min at room temperature to stop the cross-linking reaction. Cells were scraped and collected into a 50-ml polypropylene tube, and centrifuged at 3,000 rpm for 5 min at 4°C. Cells were lysed with FA cell lysis buffer with protease inhibitor, and nuclei were lysed with 1% SDS nuclear lysis buffer with protease inhibitor. Cross-linked chromatin and associated proteins were sonicated using the Bioruptor sonication device (Diagenode Inc., Denville, NJ, USA) to generate chromatin fragments with an average fragment size of 500 bp. Chromatin fragments were immunoprecipitated overnight at 4°C using control IgG or anti-STAT1 antibodies (sc-345, Santa Cruz Biotechnology, Inc., Dallas, Texas, USA) bound to Dynabeads Protein A/G magnetic beads (Life Technologies, Carlsbad, California, USA). Immunoprecipitated chromatin fragments were dissociated from the antibody-bound beads using ChIP elution buffer, cross-links were reversed by incubation with 20 mg/ml pronase for 2 h at 42°C followed by 6 h at 67°C, and DNA was purified using phenol-chloroform extraction followed by ethanol precipitation. Isolated DNA was analyzed to determine the fold enrichment of target DNA sequences relative to input chromatin. The isolated DNA was quantified by qRT-PCR analysis using SYBR Green (Kapa Biosystems, Inc., Boston, MA, USA) detection using the ABI 7900HT Fast Real-Time PCR System (Applied Biosystems, Life Technologies, Foster City, CA, USA). qRT-PCR primers for ChIP analysis are: 5′- ccctaaaggtctacccactgc-3′ (m-Irf7 Enhancer Forward) and 5′- ctccacagtcaagggttgtgt-3′ (m-Irf7 Enhancer Reverse). ChIP data were normalized to and expressed as percent of input.

### LPS Challenge Model of Septic Shock

Mice received intraperitoneal injections of LPS from *Escherichia coli* (0111:B4) in sterile PBS (30 μg/g body weight). Serum was obtained via retro-orbital bleeding 3 h after LPS administration, and stored at −80°C until analysis by ELISA. In separate experiments, survival was monitored for 72 h after LPS administration. Six to 8-week-old gender- and age-matched mice were used in all experiments.

### Statistical Analysis

Statistical analyses were performed using GraphPad Prism (GraphPad Software Inc., San Diego, California, USA). Student's *t*-test one-way analysis of variance (ANOVA) or paired *t*-tests were used as indicated in the figure legends to calculate statistical differences in mean values between groups. Results are expressed as mean ± standard deviation (SD) or mean ± standard error of the mean (SEM), as indicated in the figure legends. Values of *P* < 0.05 were considered statistically significant.

## Results

### IRF-7 Is Essential for IL-1β Production and Type I IFN Responses in a Mouse Model of Endotoxin-Induced Acute Septic Shock

**Figure 1:**
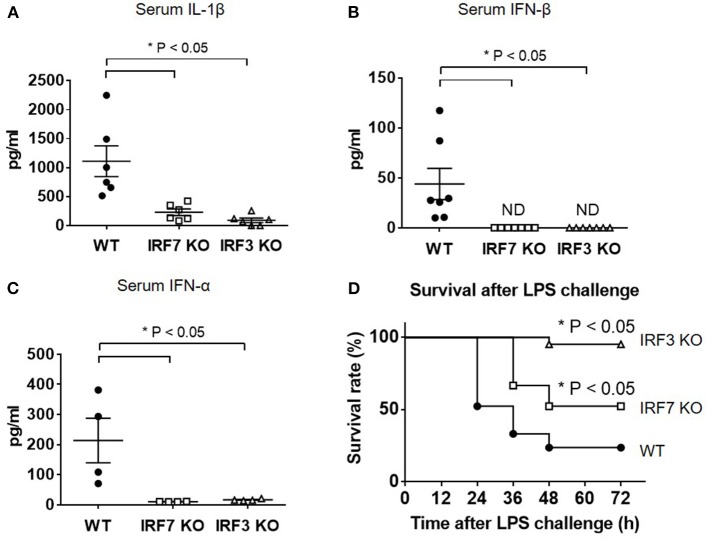
IRF-7 facilitates IL-1β and type I IFN responses to LPS *in vivo*. **(A)** ELISA analysis of IL-1β cytokine levels in serum from IRF-7 or IRF-3 knockout mice (*n* = 6) compared to wild-type control littermates (*n* = 6), 3 h after I.P. injection of 30 μg/g LPS in sterile PBS. Data are presented as mean ± SEM. One-way ANOVA was used to calculate statistical differences (*p < 0.05). **(B)** ELISA analysis of IFN-β levels in serum from IRF-7 or IRF-3 knockout mice (*n* = 7) compared to wild-type control littermates (*n* = 7), 3 h after I.P. injection of 30 μg/g LPS in sterile PBS. Data are presented as mean ± SEM. One-way ANOVA was used to calculate statistical differences (*p < 0.05). **(C)** ELISA analysis of IFN-α levels in serum from IRF-7 or IRF-3 knockout mice (*n* = 4) compared to wild-type control littermates (*n* = 4), 3 h after I.P. injection of 30 μg/g LPS in sterile PBS. Data are presented as mean ± SEM. One-way ANOVA was used to calculate statistical differences (*p < 0.05). **(D)** IRF-7 knockout mice are protected from LPS-induced endotoxin shock mortality *in vivo*. Survival of IRF-7 and IRF-3 knockout mice, compared with wild-type control littermates, following i.p. injection of 30 μg/g LPS in sterile PBS (*n* = 21 mice, *p*-value * < 0.05 compared with wild-type mice by log-rank test).

To test the hypothesis that IRF-7 is involved in the TLR-4 pathway and is required to trigger the induction of type I IFN that, in turn, drives IL-1β production *in vivo*, we challenged wild-type, *Irf7*^−/−^ and *Irf3*^−/−^ mice with a lethal dose of LPS, and measured serum IL-1β levels in wild-type and knockout mice. In accordance with other studies, wild-type mice showed a marked induction of IL-1β after intra-peritoneal LPS administration, whereas mice lacking *Irf3* exhibited severely impaired production of IL-1β (*n* = 6, *P* < 0.05 compared with wild-type mice) (Figure 1A). This is consistent with the requirement of IRF-3 for IL-1β responses to LPS administration. Interestingly, we found that serum IL-1β levels were significantly reduced in *Irf7*-knockout mice. The levels of IL-1β in *Irf7*-deficient mice were severely impaired to an extent that was similar to those in *Irf3*-knockout mice following intra-peritoneal LPS administration (*n* = 6, *P* < 0.05 compared with wild-type mice) (Figure 1A). These results suggest that TLR4-induced IL-1β production *in vivo* requires IRF-7, and is dependent on the co-operative action of both IRF-7 and IRF-3. Thus, IRF-7 is an essential factor for activation of the IL-1β response in the TLR4 pathway *in vivo*.

A recent report documented that TLR4-TRIF signaling and the IRF-3-mediated type I IFN response play important roles for *in vivo* IL-1β processing and production in response to Gram-negative bacterial infection (9). By investigating serum levels of IFN-α and IFN-β in wild-type and mutant mice following endotoxin exposure, we found that levels of type I IFN were positively correlated with levels of IL-1β in wild-type and knockout mice. As with the serum IL-1β levels (Figure 1A), wild-type mice exhibited increased type I IFN levels in serum after intra-peritoneal LPS administration, whereas serum levels of IFN-β cytokine (*n* = 7, *P* < 0.05 compared with wild-type mice) (Figure 1B) and IFN-α cytokine (*n* = 4, *P* < 0.05 compared with wild-type mice) (Figure 1C) were undetectable in both *Irf7*-knockout mice and *Irf3*-knockout mice. This suggests that, as is the case with IL-1β, IFN-β responses to LPS *in vivo* also require both IRF-7 and IRF-3.

Previous studies in mouse models of septic shock have shown that type I IFN and IL-1β contribute to LPS-induced lethality *in vivo* (27, 32–34). To assess the functional significance of attenuated type I IFN and IL-1β production in LPS-challenged *Irf7*^−/−^ and *Irf3*^−/−^ mice compared with wild-type mice, we measured the survival rate of these mice over 3 days after intra-peritoneal LPS challenge. We observed that both *Irf7*^−/−^ and *Irf3*^−/−^ mice exhibited improved survival compared with wild-type mice (*n* = 21, *P* < 0.05) (Figure 1D), thus demonstrating that both *Irf7*^−/−^ and *Irf3*^−/−^ mice exhibited increased resistance to LPS-induced endotoxin shock mortality *in vivo*. Taken together, our data demonstrate the *in vivo* physiological relevance of IRF-7 in the activation of IFN-β production by LPS, indicating that IRF-7 mediates IL-1β production *in vivo* via activation of type I IFN production, and that TLR4-induced type I IFN and IL-1β production in mice requires the combined action of both IRF-7 and IRF-3. These results provide evidence that IRF-7, which was previously shown to interact with TRIF, plays an active role in the TLR4-mediated TRIF-dependent signaling pathway.

### Macrophages, but Not Dendritic Cells, Require IRF-7, Together With IRF-3, for LPS-Stimulated IFN-β Induction

**Figure 2:**
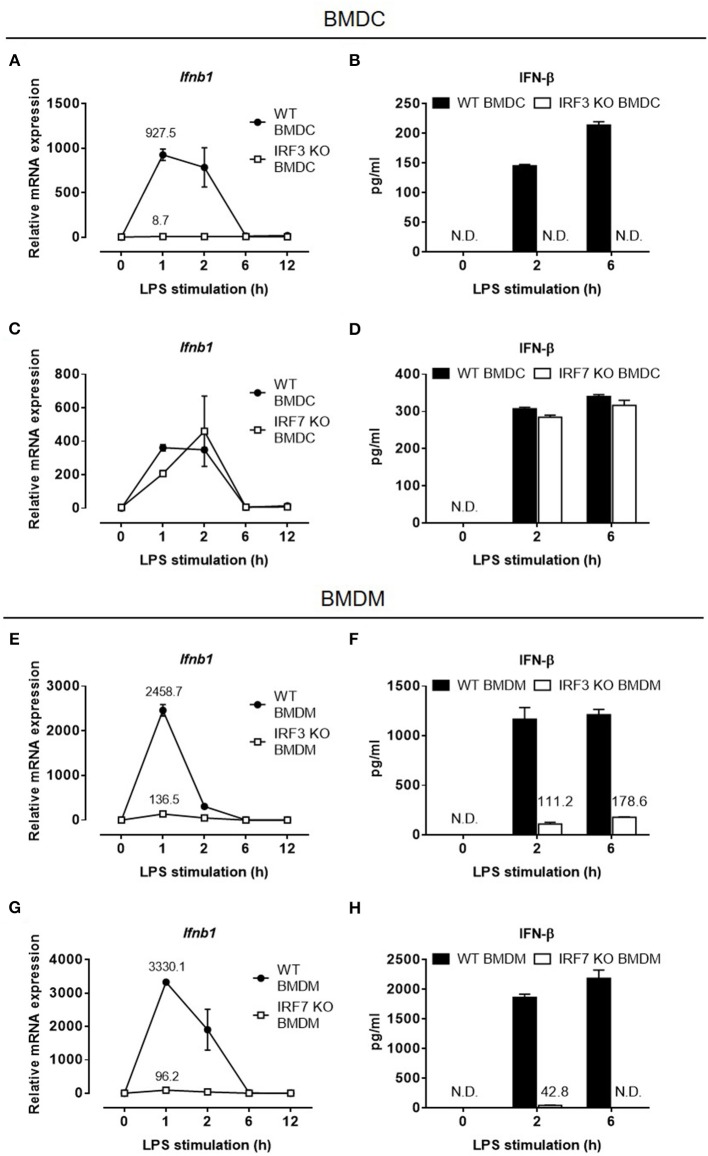
IFN-β expression in LPS-challenged macrophages depends on both IRF-7 and IRF-3, whereas IFN-β expression in LPS-challenged DCs depends on IRF-3 but not IRF-7. **(A–D)** Real-time PCR and ELISA analysis of IFN-β gene and protein expression of BMDCs from IRF-3 knockout mice **(A,B)**, and IRF-7 knockout mice **(C,D)**, together with their respective wild-type control littermates, stimulated or not with 100 ng/ml LPS for 0–12 h. *Ifnb1* expression was normalized to *Gapdh*, and expressed relative to the levels observed in un-stimulated wild-type control cells. Data are presented as mean ± SD of duplicate determinations from one representative of at least two independent experiments (N.D.: not detected). **(E–H)** Real-time PCR and ELISA analysis of IFN-β gene and protein expression of BMDMs from IRF-3 knockout mice **(E,F)**, and IRF-7 knockout mice **(G,H)**, together with their respective wild-type control littermates, stimulated or not with 100 ng/ml LPS for 0–12 h. *Ifnb1* expression was normalized to *Gapdh* and expressed relative to the levels observed in un-stimulated wild-type control cells. Data are presented as mean ± SD of duplicate determinations from one representative of at least three independent experiments (N.D.: not detected).

The loss of type I IFN production in *Irf7*-deficient mice after LPS administration was intriguing. This is because previous studies by Honda et al. have, unequivocally, demonstrated that IFN-β mRNA transcription is largely retained in LPS-stimulated *Irf7*-deficient DCs, but is markedly abolished in *Irf3*-deficient DCs (24). We sought to confirm these findings by analyzing IFN-β mRNA induction and protein secretion in BMDCs from *Irf7*- and *Irf3*-knockout mice. As in the previous report by Honda et al. BMDCs from *Irf7*-knockout mice produced relatively normal amounts of IFN-β at both mRNA and protein levels, whereas IFN-β gene induction and protein secretion were inhibited in LPS-stimulated *Irf3*-knockout BMDCs (Figures 2A–D). Hence, our results are consistent with the previous study by Honda et al. which concluded that activation of the type I IFN response in LPS-stimulated DCs depended entirely on IRF-3 (24, 27).

Although both macrophages and DCs secrete IFN-β after LPS stimulation, BMDMs consistently produced significantly higher levels of IFN-β than DCs in response to a similar LPS stimulation (Supplementary Figure 1). Because IRF-7 has previously been shown to act together with IRF-3 to induce later-phase production of high levels of type I IFN in fibroblasts during viral infections (35), we hypothesized that in macrophages, which produced higher levels of IFN-β compared with DCs, IRF-7, in addition to IRF-3, induces IFN-β production after LPS stimulation. To test this hypothesis, we evaluated both IFN-β mRNA induction and protein secretion in BMDMs derived from *Irf7*- and *Irf3*-knockout mice. In line with previous studies, IFN-β production was impaired in BMDMs lacking *Irf3* (Figures 2E,F), indicating that IRF-3 is critical for type I IFN production in macrophages. Surprisingly, unlike in BMDCs, LPS-induced IFN-β expression in *Irf7*-deficient BMDMs was markedly inhibited (Figures 2G,H), suggesting that, unlike in BMDCs, IRF-7 is critical for TLR4-mediated IFN-β induction in macrophages. Therefore, our findings suggest that, as in viral-infected fibroblasts, induction of type I IFN in the TLR4 pathway in macrophages also depends on both IRF-7 and IRF-3 activities.

### IRF-7 Is Constitutively Expressed in Resting Bone Marrow-Derived Macrophages, but Not in Dendritic Cells

**Figure 3:**
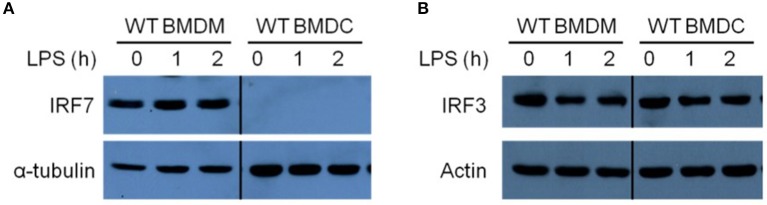
Resting macrophages, but not DCs, constitutively express IRF-7 protein. Western immunoblot analysis of total IRF-7 **(A)** and total IRF-3 **(B)** protein expression in whole cell lysates of wild-type BMDMs and BMDCs, stimulated or not with 100 ng/ml LPS for 0–2 h. Data are representative of at least three independent experiments.

IRF-7 is constitutively expressed in pDCs, where it is critical for rapid and robust type I IFN production during viral infections (24). The involvement of IRF-7 in the regulation of TLR4-induced IFN-β production in BMDMs led us to hypothesize that, as in pDCs, BMDMs may also constitutively express IRF-7 and this may be responsible for the robust activation of IFN-β production in these cells after LPS stimulation. To investigate this possibility, we analyzed the expression of IRF-7 protein in resting BMDMs and BMDCs by Western blotting. In line with the lack of IRF-7 function during induction of IFN-β production in DCs, we did not observe any IRF-7 protein in resting wild-type BMDCs (Figure 3A). On the contrary, resting wild-type BMDMs constitutively expressed IRF-7 protein (Figure 3A), as was also the case in pDCs. On the other hand, as expected, IRF-3 protein is constitutively expressed in both macrophages and DCs (Figure 3B). The kinetics of IRF-3 phosphorylation in response to LPS treatment was comparable between wild-type BMDMs and BMDCs (Supplementary Figure 2).

IRF-7 is an IFN-inducible protein, up-regulated by autocrine signaling through the IFN-α/β receptor (IFNAR), that promotes further production of type I IFN after viral infection in fibroblasts (24, 35–37). In contrast, we found that IRF-7 expression in macrophages remained constant up to 2 h after LPS stimulation, which corresponds with the peak in IFN-β transcriptional induction (Figure 3A). This suggests that pre-existing IRF-7 protein is responsible for the activation of type I IFN induction in macrophages. It is worth mentioning that IRF-7 expression in DCs remained undetectable within the first 2 h post-LPS stimulation. Altogether, these data suggest that, as observed in pDCs, resting BMDMs possess a pre-existing pool of constitutively expressed IRF-7 protein that is necessary for the activation of robust IFN-β responses in macrophages after LPS stimulation.

### Basal Type I IFN Signaling Sustains Constitutive IRF-7 Expression, and Is Required for LPS-Stimulated IFN-β Induction in Macrophages

**Figure 4:**
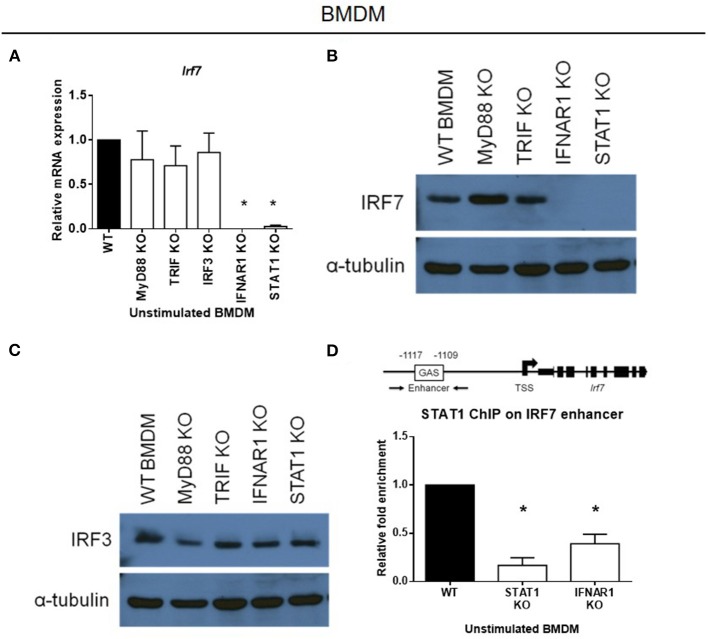
Constitutive expression of IRF-7 in resting macrophages is sustained by constitutive IFNAR signaling and STAT1 binding to the *Irf7* enhancer. **(A)** Real-time PCR analysis of *Irf7* gene expression in resting BMDMs from MyD88, TRIF, IRF-3, IFNAR1, and STAT1 knockout mice, compared to wild-type control littermates. *Irf7* expression was normalized to *Gapdh*, and expressed relative to the levels observed in un-stimulated wild-type control cells. Data are presented as mean ± SEM of at least three independent experiments. One-way ANOVA was used to calculate statistical differences (*p < 0.05). **(B,C)** Western immunoblot analysis of total IRF-7 **(B)** and total IRF-3 **(C)** protein expression in whole cell lysates of resting BMDMs from MyD88, TRIF, IFNAR1, and STAT1 knockout mice, compared to wild-type control littermates. Data are representative of at least two independent experiments. **(D)** ChIP analysis of STAT1 binding at the IRF-7 enhancer in resting BMDMs from STAT1 and IFNAR1 knockout mice compared to wild-type control littermates. ChIP-enriched DNA was normalized to input DNA and expressed relative to the levels observed in STAT1 ChIP in un-stimulated wild-type control cells. Data shown are presented as mean ± SEM of at least three independent experiments. One-way ANOVA was used to calculate statistical differences (*p < 0.05).

As IRF-7 is already constitutively expressed in macrophages, we hypothesized that this was due to the presence of a basal type I IFN production and signaling in macrophages, that is not present in DCs. To check this hypothesis, we analyzed the expression of the IRF-7 protein in BMDMs prepared from mice with defective type I IFN signaling components, namely *Ifnar1* and *Stat1*. We found that basal IRF-7 mRNA and protein levels were markedly inhibited in resting BMDMs lacking either *Ifnar1* or *Stat1* (Figures 4A,B), whereas IRF-3 protein levels remained unaffected (Figure 4C). However, constitutive IRF-7 expression at both mRNA and protein levels were found to be largely unaltered in BMDMs lacking other components of TLR signaling, namely *Myd88, Trif* and *Irf3* (Figures 4A,B). Thus, our data suggest that constitutive IRF-7 expression in resting BMDMs is mediated by basal type I IFN signaling in a STAT1-dependent manner.

Given that basal type I IFN signaling regulates constitutive IRF-7 expression in BMDMs, our next step was to check whether the absence of IRF-7 protein in resting BMDCs was due to an absence of basal type I IFN production and signaling in these cells. To do so, we analyzed the basal levels of IFN-β expression in resting BMDCs and BMDMs. Our results show that basal expression of IFN-β mRNA was significantly lower in wild-type BMDCs than in wild-type BMDMs (Supplementary Figure 3A), suggesting that DCs, intrinsically, lack basal type I IFN production and signaling, which explains the complete absence of IRF-7 protein in these cells. In line with the absence of type I IFN production and signaling, resting wild-type BMDCs were found to express minimal amounts of IRF-7 mRNA (over 200-fold lower than BMDMs), whereas BMDMs expressed high levels of *Irf7* transcripts (Supplementary Figure 3B). Overall, our data indicate that constitutive *Irf7* expression in macrophages is primarily regulated at the transcriptional level by basal IFN-β production and type I IFN signaling, and that this is not present in DCs.

Type I IFN signaling is mediated by STAT1 activation via the IFNAR. To understand how *Irf7* is constitutively regulated at the transcriptional level in macrophages, we explored whether STAT1 regulates the transcription of *Irf7* directly by basal type I IFN signaling. The murine *Irf7* enhancer contains a IFN-γ-activated site (GAS) sequence, which binds STAT1 at a site 1.1 kb upstream of the transcription start site (TSS) (38). Chromatin immunoprecipitation (ChIP) experiments on STAT1 binding at this upstream GAS enhancer show significant constitutive binding of STAT1 to the *Irf7* enhancer region in resting wild-type macrophages. This constitutive binding of STAT1 to the *Irf7* enhancer region was completely abrogated in resting *Ifnar1*-deficient BMDMs that had disrupted basal type I IFN signaling (Figure 4D). These results indicate that basal IFN-β production and signaling in resting macrophages results in constitutive STAT1 binding to the *Irf7* enhancer region, and this sustains constitutive *Irf7* transcription and subsequent protein expression.

**Figure 5:**
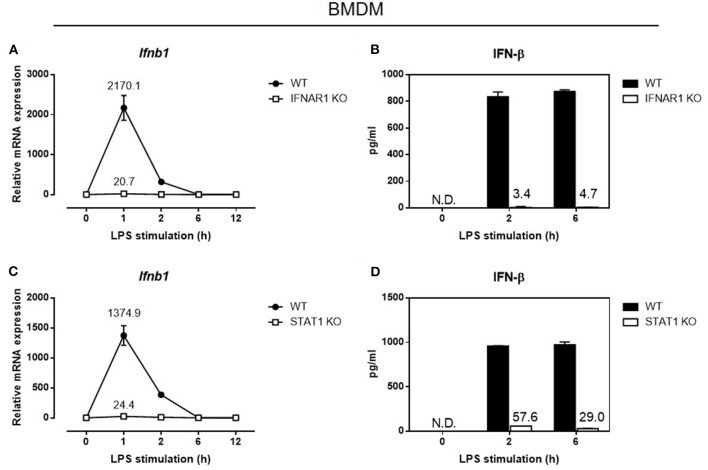
Autocrine/paracrine IFNAR1-STAT1 signaling is required for IFN-β gene and protein expression in LPS-challenged macrophages. Real-time PCR and ELISA analysis of IFN-β gene and protein expression of BMDMs from IFNAR1 knockout mice **(A,B)**, and STAT1 knockout mice **(C,D)**, compared to wild-type control littermates, stimulated or not with 100 ng/ml LPS for 0–12 h. *Ifnb1* expression was normalized to *Gapdh* and expressed relative to the levels observed in un-stimulated wild-type control cells. Data are presented as mean ± SD of duplicate determinations from one representative of at least three independent experiments (N.D.: not detected).

The absence of constitutive IRF-7 expression in resting macrophages lacking *Ifnar1* or *Stat1* implies that macrophages with defective type I IFN signaling would display defective induction of IFN-β in response to LPS. Consistent with our observations in *Irf7*-deficient macrophages, IFN-β mRNA induction and protein secretion were markedly abolished in LPS-stimulated macrophages that had defective type I IFN signaling, namely *Ifnar1*- and *Stat1*-deficient BMDMs (Figures 5A–D). Although type I IFN signaling following IFNAR engagement can also be mediated by STAT3 homodimers, we found that LPS-stimulated IFN-β expression was not affected in *Stat3*-knockout BMDMs, in contrast to *Ifnar1*- and *Stat1*-deficient BMDMs. On the contrary, IFN-β expression in response to LPS was elevated in *Stat3*-knockout BMDMs compared with wild-type BMDMs (Supplementary Figure 4). Therefore, our data indicate that STAT1, but not STAT3, downstream of basal type I IFN signaling, mediates constitutive IRF-7 expression in resting macrophages, which is in turn required for IFN-β responses in LPS-stimulated macrophages.

The absence of constitutive IRF-7 expression and the lack of basal type I IFN production and signaling in resting DCs prompted us to speculate that DCs with defective type I IFN signaling would display normal levels of IFN-β induction in response to LPS, similar to *Irf7*-deficient DCs. Indeed, in contrast to LPS-stimulated BMDMs, IFN-β induction in LPS-stimulated BMDCs lacking *Ifnar1* was not affected (Supplementary Figure 5). This confirms that TLR4-induced IFN-β production in DCs is independent of constitutive type I IFN signaling.

### LPS-Induced IRF-3 Phosphorylation and Nuclear Translocation in Macrophages Is Not Affected by the Absence of IRF-7

**Figure 6:**
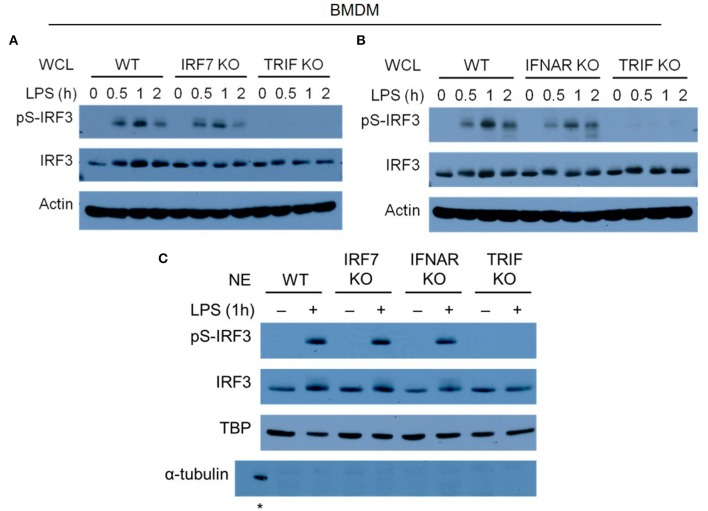
IRF-7 and autocrine/paracrine IFNAR signaling regulate IFN-β expression independent of IRF-3 phosphorylation in LPS-challenged macrophages. **(A,B)** Western immunoblot analysis of phospho-IRF-3 and total IRF-3 protein expression in whole cell lysates (WCL) of IRF-7 knockout **(A)** or IFNAR1 knockout **(B)** BMDMs, compared to wild-type (WT) and TRIF knockout BMDMs, stimulated or not with 100 ng/ml LPS for 0–2 h. Data are representative of at least three independent experiments. Actin was used as a loading control. **(C)** Western immunoblot analysis of phospho-IRF-3 and total IRF-3 protein expression in nuclear extracts (NE) of wild-type (WT), IRF-7 knockout, IFNAR1 knockout, and TRIF knockout BMDMs, stimulated or not with 100 ng/ml LPS for 0–2 h. Data are representative of at least two independent experiments. Actin and TATA-binding protein (TBP) were used as loading controls. *indicates α-tubulin detected in WCL of un-stimulated WT BMDMs as a control.

IRF-3 phosphorylation and nuclear translocation is necessary for the activation of type I IFN production by macrophages after LPS stimulation (14, 39–41). Following our finding that both IRF-7 and IRF-3 are required for IFN-β induction in macrophages and in mice, we studied whether they could affect each other's phosphorylation and nuclear translocation in response to bacterial LPS. To do so, we performed biochemical analyses to determine the phosphorylation and nuclear translocation of IRF-3 in *Irf7*-deficient BMDMs before and after LPS stimulation. Our results demonstrated that IRF-3 phosphorylation in LPS-stimulated *Irf7*-knockout BMDMs was not much different from that in wild-type BMDMs (Figure 6A). The nuclear translocation of the phosphorylated form of IRF-3 is critical for the activation of *Ifnb* in LPS-challenged macrophages. Our analyses show that nuclear extracts from LPS-stimulated *Irf7*-deficient BMDMs and wild-type BMDMs contained a similar amount of IRF-3 (Figure 6C). We also found relatively normal levels of IRF-3 phosphorylation and nuclear translocation in LPS-stimulated BMDMs lacking *Ifnar1* when compared with that in wild-type BMDMs (Figures 6B,C). This supports the concept that constitutive type I IFN signaling is necessary for constitutive IRF-7 expression in macrophages, but is dispensable for IRF-3 phosphorylation and nuclear translocation in these cells. As expected, LPS-stimulated *Trif*-knockout BMDMs showed severe impairment in both phosphorylation and nuclear translocation of IRF-3 (Figures 6A–C). Due to the lack of a reliable antibody specific against the endogenous phosphorylated form of IRF-7, we were unable to investigate IRF-7 phosphorylation and nuclear translocation in LPS-stimulated *Irf3*-deficient macrophages. Thus, we concluded that TRIF-mediated phosphorylation and nuclear translocation of IRF-3 is completely independent of IRF-7 activity in LPS-stimulated macrophages. Taken together with our finding that IRF-7 levels are largely unaltered in IRF-3-null macrophages (Supplementary Figure 6), these data suggest that IRF-7 and IRF-3 are both required in combination to achieve optimal IFN-β production in endotoxin-challenged macrophages.

### IRF-7-Mediated IFN-β Induction in LPS-Stimulated Macrophages Depends on TRIF and TBK-1

**Figure 7:**
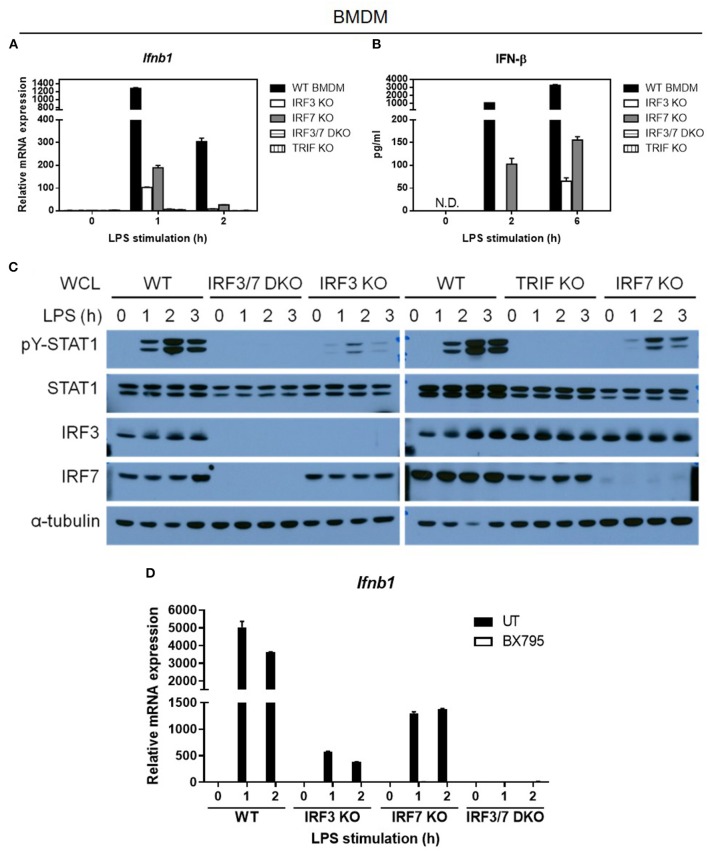
TBK1 is required for the activation of both IRF-7 and IRF-3 downstream of TRIF for optimal IFN-β expression in LPS-challenged macrophages. **(A,B)** Real-time PCR and ELISA analysis of IFN-β gene and protein expression of BMDMs from IRF-3, IRF-7, and TRIF single knockout mice, and IRF-3-IRF-7 double knockout mice, compared to wild-type control littermates, stimulated or not with 100 ng/ml LPS for the indicated times. *Ifnb1* expression was normalized to *Gapdh* and expressed relative to the levels observed in un-stimulated wild-type control cells. Data are presented as mean ± SD of duplicate determinations from one representative of at least two independent experiments (N.D.: not detected). **(C)** Western immunoblot analysis of phospho-STAT1 and total STAT1 protein expression in whole cell lysates of BMDMs from IRF-3, IRF-7, and TRIF single knockout mice, and IRF-3-IRF-7 double knockout mice, compared to wild-type control littermates, stimulated or not with 100 ng/ml LPS for 0–6 h. Data are representative of at least two independent experiments. **(D)** Real-time PCR analysis of IFN-β gene expression in BMDMs from IRF-3 single knockout mice, IRF-7 single knockout mice, and IRF-3-IRF-7 double knockout mice, compared to wild-type control littermates, pre-treated or not with 2 μM BX795 (TBK1 inhibitor) for 1 h, and then stimulated or not with 100 ng/ml LPS for 0–2 h. *Ifnb1* expression was normalized to *Gapdh* and expressed relative to the levels observed in un-treated and un-stimulated wild-type control cells. Data are presented as mean ± SD of duplicate determinations from one representative of at least two independent experiments.

As IRF-3 alone can mediate type I IFN induction in BMDCs after LPS stimulation, and LPS-stimulated *Irf7*-deficient macrophages showed normal phosphorylation and nuclear translocation of IRF-3, we speculated that the presence of IRF-3 alone in *Irf7*-deficient macrophages might still retain some ability to mediate IFN-β induction, despite the absence of IRF-7 in these cells. Indeed, LPS-induced *Irf7*-deficient macrophages can still produce IFN-β, although its levels were low and attenuated compared with the ones found in wild-type BMDMs (Figures 7A,B). Our results indicated that *Irf3*-deficient macrophages also expressed low levels of *Ifnb* transcripts (Figures 7A,B). Consistent with these data, LPS-stimulated macrophages lacking either *Irf7* or *Irf3* exhibited low levels of STAT1 phosphorylation (Figure 7C). Thus, these results further support the premise that IRF-7 and IRF-3 activation and nuclear translocation in LPS-stimulated macrophages are independent processes. Our data also indicate that IRF-7 or IRF-3 alone can mediate IFN-β induction by LPS, but when IRF-7 and IRF-3 are simultaneously present in macrophages, transactivation of *Ifnb* is markedly enhanced, which, according to our results, is a requirement for robust IL-1β production in mice after LPS challenge.

The signaling adaptor MyD88 has been demonstrated to activate IRF-7 for induction of type I IFN by pDCs in response to virus infection and TLR7/9 activation (24, 42). However, this and other studies have shown that IFN-β expression was not impaired in MyD88-deficient BMDMs compared with wild-type BMDMs after LPS stimulation (Supplementary Figure 7). These results indicate that, unlike the requirement for MyD88 and IRF-7 in TLR7/9-activated pDCs, IFN-β induction in TLR4-activated macrophages is dependent on IRF-7, but is independent of MyD88. Previous biochemical studies have demonstrated that TRIF can interact with and activate both IRF-7 and IRF-3 *in vitro* (25, 26). Hence, we predicted a complete loss of IFN-β induction by LPS in macrophages prepared from mice lacking *Trif* or both *Irf3* and *Irf7*, if it is true that TRIF is required for activation of both IRF-7 and IRF-3. As expected, LPS-stimulated IFN-β mRNA induction and protein secretion was completely abolished in macrophages lacking *Trif* , thereby suggesting that TRIF promotes the activation of both IRF-7 and IRF-3 in the TLR4 pathway (Figures 7A–C). Correspondingly, as in *Trif*-deficient macrophages, we also found that IFN-β transcription and secretion were entirely abrogated in *Irf3*/*Irf7* double deficient BMDMs (Figures 7A–C), supporting the hypothesis that TRIF mediates the activation of both IRF-7 and IRF-3 in LPS-stimulated macrophages.

Given that TRIF can interact with both IRF-7 and IRF-3, and *in vitro* kinase assays have shown that TBK-1 can mediate IRF-3 and IRF-7 phosphorylation (43–47), we hypothesized that TBK-1 can mediate IRF-7 activation in LPS-stimulated macrophages. To test this hypothesis, we used BX795, a small molecule inhibitor of TBK-1, to test whether IRF-7 activity and IRF-7-mediated type I IFN production are also dependent on TBK-1 activity (48, 49). Due to the lack of a reliable antibody specific against the endogenous phosphorylated form of IRF-7, we measured LPS-induced IFN-β gene expression in *Irf3* single knockout, *Irf7* single knockout, and *Irf3/Irf7* double knockout BMDMs in the absence or presence of BX795 (Figure 7D and Supplementary Figure 8). In line with previous studies, BX795 inhibited the IRF-3-mediated IFN-β transcription present in *Irf7*-knockout BMDMs. Interestingly, we found that BX795 also inhibited the IRF-7-mediated IFN-β transcription present in *Irf3*-knockout BMDMs, suggesting that TBK-1 also mediates IRF-7 activation and IRF-7-mediated type I IFN production in LPS-stimulated macrophages. Moreover, we also found that BX795 completely inhibited IFN-β transcription in wild-type BMDMs, similar to levels seen in *Irf3*/*Irf7* double knockout BMDMs, rather than to levels seen in the single knockout BMDMs, suggesting that TBK-1 is the kinase that mediates the phosphorylation of both IRF-3 and IRF-7 in TLR4 signaling in macrophages. In summary, we conclude that TRIF mediates activation of both IRF-7 and IRF-3 via TBK-1 in the macrophage TLR4 pathway.

## Discussion

**Figure 8:**
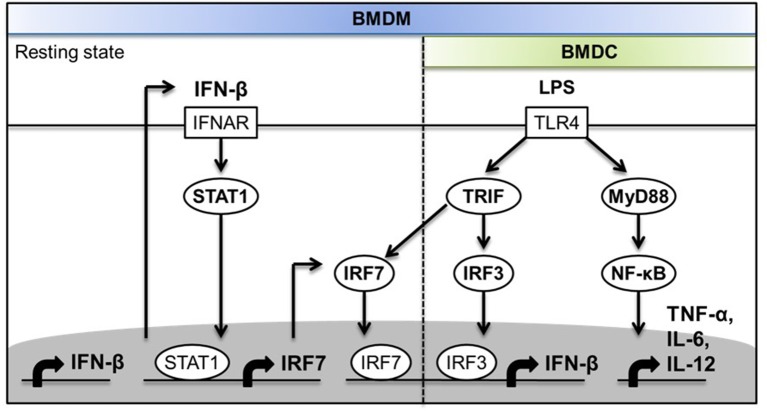
Schematic diagram depicting the molecular mechanisms of the involvement of IRF-7 in TLR4-induced IFN-β expression in macrophages but not in DCs.

Type I IFN is necessary for IL-1β production by the non-canonical NLRP3 inflammasome in response to Gram-negative bacterial infection. TRIF is essential for non-canonical NLRP3 inflammasome activation by LPS of Gram-negative bacteria through the activation of type I IFN induction (9). Previous studies have demonstrated that LPS induces type I IFN production via the TLR4-TRIF-TBK-1-IRF-3 pathway to promote the transcriptional induction of *Casp11*, which encodes caspase-11 as the key mediator of the non-canonical NLRP3 inflammasome (9). As TRIF was previously shown to interact with IRF-7 (25), and IRF-7 is known as a “master regulator” of type I IFN responses in viral infections (24, 35, 36), we hypothesized that IRF-7 is specifically involved in the TLR4 pathway and is required to trigger the induction of TRIF-dependent type I IFN that, in turn, drives IL-1β production. We tested this hypothesis in an established mouse model of endotoxin shock, in which it has been previously shown that the induction of IL-1β responses *in vivo* is dependent on the activation of type I IFN production by TLR4 in a TRIF- and IRF-3-dependent manner (7–9). By using this animal model, we identified IRF-7 as an essential regulator of IL-1β and type I IFN production in mice. We also demonstrated that the production of type I IFN and IL-1β in mice is dependent on the combined action of both IRF-7 and IRF-3, which have been shown to interact with TRIF in a yeast two-hybrid screening study (25). The low amounts of residual IL-1β present in the serum of IRF-7 knockout mice might be due to the activation of non-canonical inflammasome by “cytosolic” LPS (7, 8). Macrophages and DCs are key antigen-presenting cells that trigger both pro-inflammatory cytokine production and type I IFN production in the innate immune response to LPS. Our results show for the first time that macrophages, but not DCs, constitutively express IRF-7 and require IRF-7 to promote robust IFN-β induction following LPS stimulation. Macrophages lacking either *Irf7* or *Irf3* produce significantly lower levels of type I IFN in response to LPS, which has led us to propose a new paradigm whereby both IRF-7 and IRF-3 are essential for TLR4-induced IFN-β production in macrophages. The activation of IRF-7 and IRF-7-mediated IFN-β induction in macrophages is dependent on TBK-1, which has been shown to be activated by TRIF in response to TLR4 ligation by LPS (43–46, 48–51). In contrast, we found that DCs lack constitutive IRF-7 expression, and are dependent on IRF-3, but not IRF-7, for IFN-β induction following LPS stimulation in the TLR4 pathway. Our results indicate that cell-type specific basal type I IFN production and signaling present in resting macrophages, but absent in DCs, is largely responsible for constitutive IRF-7 expression at both mRNA and protein levels, which, in turn, is required for IFN-β responses in LPS-stimulated macrophages, but not in DCs (Figure 8). Taken together, our *in vitro* studies in macrophages and DCs suggest that macrophages may represent a key cell type that contributes to type I IFN and IL-1β responses *in vivo*, since they depend on both IRF-7 and IRF-3 activities for activation of type I IFN responses after LPS stimulation.

### Constitutively Expressed IRF-7 in Resting Macrophages act Together With IRF-3 to Confer Rapid and Robust IFN-β Induction in the TLR4 Pathway

In this study, we showed that IL-1β is rapidly induced in mice during LPS challenge. IL-1β has been shown to play an important role in early host defense against bacterial infections. Type I IFN is also rapidly induced in response to LPS and is essential for activation of IL-1β production by the non-canonical caspase 11-dependent inflammasome in mice. The timely and robust production of IFN-β may possibly contribute to the kinetics and amounts of IL-1β production by macrophages during Gram-negative bacteria infection. Type I IFN production differs in kinetics and magnitude between cell types (32, 52–55). We and others have previously reported that human monocytes produced IFN-β within 1–2 h of exposure to Sendai virus or LPS, whereas non-myeloid cell types, such as HeLa cells and fibroblasts, produced IFN-β after 6 h post-infection (52, 53, 56, 57). Maniatis et al. have used virus-infected human epithelial HeLa cells as a model to identify component transcription factors of the virus-induced IFN-β “enhanceosome,” such as NF-κB RelA/p50, IRF-3/7, and ATF-2/c-Jun, that act at the IFN-β enhancer to induce IFN-β transcription (58). On the other hand, we showed that the rapid induction of IFN-β transcription in human monocytes was determined to some extent by the constitutive binding of the myeloid-specific transcription factors PU.1 and IRF-8 to the enhancer region of the *IFNB* promoter, which promoted the recruitment of IRF-3 to the *Ifnb* locus through direct physical interaction between IRF-8 and IRF-3 (52). We also noticed that, similar to human monocytes, murine BMDMs also rapidly expressed IFN-β mRNA within 1–2 h of LPS exposure. Since PU.1 and IRF-8 are present and functionally important in myeloid cells, it is highly plausible that in BMDMs, IRF-8 and PU.1 also constitutively bind to the *Ifnb* promoter and facilitate the recruitment of the transcription factors IRF-7 and IRF-3 to induce the rapid and robust LPS-induced IFN-β gene transcription in macrophages. The transcription factor IRF-7 is an IFN-inducible protein and is typically not endogenously expressed in most cell types except pDCs. The constitutive expression of IRF-7 protein in pDCs was previously shown to be responsible for high-level and rapid IFN-α production by these cells after stimulation with TLR7/8/9 ligands (24, 42). In contrast, the late inducible expression of IRF-7 in fibroblasts after virus infection was shown to be responsible for a delayed kinetics of type I IFN production (35, 36, 59–61). In the present study, when analyzing the expression of IRF-7 in BMDMs, we found, to our surprise, that resting BMDMs already expressed significant amounts of IRF-7 protein, a protein that is absent in resting BMDCs. Macrophages from mice lacking *Irf7* showed a severe decrease in IFN-β production, suggesting that constitutive expression of IRF-7 in macrophages is responsible for the rapid and robust activation of the *Ifnb* promoter. This is consistent with defects in IFN-β production in BMDMs from mice lacking *Ifnar1* or *Stat1*, which lack constitutive expression of IRF-7. Altogether, our studies suggest that the rapid and robust activation of IFN-β production in macrophages is likely determined by a combination of both an already primed *Ifnb* promoter with constitutively bound PU.1 and IRF-8, and the constitutively expressed IRF-7 and IRF-3, which were rapidly activated and recruited to the primed enhancer region of the *Ifnb* promoter in macrophages after LPS stimulation.

### Involvement of Both IRF-7 and IRF-3 in the Activation of Type I IFN Induction in Macrophages Suggests the Use of TRIF, Rather Than MyD88, in the TLR4 Signaling Pathway

Our discovery that IRF-7, in concert with IRF-3, regulates LPS-induced type I IFN production in mice and in macrophages, but not in DCs, provides a possible molecular explanation for the preferential usage of the TRIF rather than the MyD88 adaptor, in TLR4-induced type I IFN production. The transcription factors IRF-7 and IRF-3 are key master regulators of type I IFN production during viral infection or after activation by TLR ligands. Activation of these transcription factors in the TLR pathways is primarily mediated by two main adaptors: MyD88 and TRIF. Different adaptors engage different transcription factors that may dictate the kinetics, magnitude, and/or types of type I IFN genes expressed. MyD88 is utilized by all TLRs except TLR3, whereas TRIF is only used by TLR3 and TLR4 (62). TLR4 is the only TLR that can separately trigger pro-inflammatory cytokines and type I IFN responses, which have previously been demonstrated to be mediated by MyD88 and TRIF, respectively. Biochemical co-immunoprecipitation assays and FRET microscopy in live cells have been used to demonstrate that MyD88 directly interacts with IRF-7, but not with IRF-3 (42, 63). This MyD88-IRF-7 pathway was found to operate mainly in pDCs, and is largely responsible for the rapid induction of high levels of type I IFN, following the activation of TLR7/8/9 by nucleic acids during viral infection. The induction of type I IFN by TLR7/8/9 ligation was defective in splenic pDCs prepared from mice lacking *MyD88* or *Irf7*, but not *Irf3*, which was consistent with the ability of MyD88 to physically associate with IRF-7, but not with IRF-3 (24, 42). These studies clearly demonstrate that direct interactions between IRF-7 and MyD88 are essential for TLR7/8/9-induced type I IFN production in pDCs (63, 64).

While MyD88 forms a complex with only IRF-7, the adaptor protein TRIF, in contrast, has been shown to interact with and activate both IRF-7 and IRF-3 *in vitro* (25, 26), suggesting that the transcriptional activation of type I IFN genes, such as *Ifnb*, after TLR4 ligation by LPS may be regulated by both IRF-7 and IRF-3 via TRIF. However, in transient transfection studies, overexpression of IRF-3 alone was sufficient to induce the activation of the *Ifnb* promoter (65). Moreover, BMDCs from mice lacking *Irf7* displayed normal LPS-stimulated IFN-β transcription, whereas *Irf3*^−/−^ BMDCs lacked LPS-stimulated IFN-β induction (24). Therefore, the general consensus is that the transcription factor IRF-3, rather than IRF-7, is the only mediator of IFN-β expression in the TLR4 pathway. There is also the general assumption that IRF-3 is the only factor that is responsible for the induction of IL-1β production in mice via type I IFN production in the non-canonical NLRP3 inflammasome pathway after *in vivo* Gram-negative bacteria infection. In our present study, we surprisingly found that in addition to IRF-3, the transcription factor IRF-7 is essential for type I IFN induction in mice and in macrophages. Moreover, we also demonstrated that, as with IRF-3, IRF-7 activation and IRF-7-mediated IFN-β production are also dependent on the adaptor TRIF in the TLR4 pathway. Our analysis of IRF-3 single knockout and IRF-7 single knockout BMDMs, together with IRF-3/IRF-7 double knockout and TRIF knockout BMDMs, showed that the absence of either IRF-3 or IRF-7 results in weak LPS-induced IFN-β responses, while the absence of both IRF-3 and IRF-7 phenocopies the complete shutdown of the LPS-induced IFN-β response as observed in TRIF knockout BMDMs. This suggests that both IRF-3 and IRF-7 need to act together downstream of TRIF to induce optimal IFN-β expression in LPS-challenged macrophages. Furthermore, we showed that macrophages, but not DCs, are dependent on both IRF-7 and IRF-3 to activate the *Ifnb* promoter. The requirement for both IRF-7 and IRF-3 in the activation of type I IFN production in macrophages by LPS could be a possible explanation for the preferential use of TRIF, rather than MyD88, in the TLR4 response to LPS, because of the ability of TRIF to interact with and activate both IRF-7 and IRF-3, whereas MyD88 can associate with IRF-7 but not with IRF-3. Thus, to the best of our knowledge, our study is the first to demonstrate that the physical interaction of TRIF with both IRF-7 and IRF-3 is functionally required for robust induction of type I IFN in macrophages. Additionally, mice lacking either *Irf7* or *Irf3* exhibited severely impaired IL-1β production *in vivo* after LPS challenge, indicating that IL-1β production in mice by LPS requires optimal type I IFN production, which is mediated by both IRF-7 and IRF-3 via the TRIF pathway.

TRIF has been shown to form a complex with TBK-1, a protein kinase that has been reported to directly phosphorylate IRF-3 and IRF-7 in response to viral infection or TLR3 and TLR4 stimulation based on *in vitro* kinase assays (43–47). Recent evidence has demonstrated that TRIF-dependent signaling cascades in LPS-stimulated macrophages involve the recruitment and phosphorylation of TBK1 at Ser172, of TRIF at the pLxIS motif, and of IRF-3 at Ser396 at the endosomal compartment (43–46, 48–51). Our finding that IRF-7 is critical for IFN-β induction in LPS-stimulated macrophages prompted us to investigate the contribution of TBK-1 to the activation of IRF-7 and IRF-7-mediated type I IFN production by LPS. Due to the lack of a reliable antibody specific against the endogenous phosphorylated form of IRF-7, we could only assess the involvement of TBK-1 on IRF-7 activity by measuring IFN-β expression in LPS-stimulated *Irf3*-deficient macrophages after treatment with BX-795, which is a specific inhibitor of TBK-1. The BX-795 inhibition of TBK-1 in macrophages lacking either *Irf3* or *Irf7* can strongly abrogate the remaining type I IFN production in the single knockout BMDMs, suggesting that, similar to IRF-3, the transcription factor IRF-7 is also activated by TBK-1 and mediates type I production through a TRIF-induced TBK-1-dependent pathway in LPS-TLR4 signaling. Thus, our study further strengthened the concept that TRIF, rather than MyD88, is the preferred adaptor to mediate type I IFN induction in the TLR4 pathway in macrophages, because TRIF is endowed with the ability to activate both IRF-7 and IRF-3 via the recruitment of TBK-1, which was previously shown to phosphorylate both IRF-7 and IRF-3.

In summary, we have identified that IRF-7 plays an essential role in the production of type I IFN in the TLR4 signaling pathway. Importantly, we showed that IL-1β and IFN-β production in LPS-challenged mice requires the concerted activation of both IRF-7 and IRF-3 via a TRIF-TBK-1 signaling pathway. Furthermore, we demonstrated that macrophages, but not DCs, are dependent on both IRF-7 and IRF-3 for robust activation of IFN-β production. This suggests that macrophages are an important source of IFN-β that may contribute to the activation of IL-1β production by the non-canonical inflammasome pathway *in vivo* following LPS administration. These novel mechanistic insights into the molecular basis of the divergent roles played by macrophages and DCs in anti-microbial immunity will critically inform future studies of their disparate roles in host protection against bacterial pathogens.

## Data Availability Statement

All datasets generated for this study are included in the article/Supplementary Material.

## Ethics Statement

The animal study was reviewed and approved by Biological Resource Centre (BRC) Institutional Animal Care and Use Committee (IACUC).

## Author Contributions

W-XS designed and performed experiments, acquired and analyzed data, and wrote the manuscript. JY designed and performed experiments, acquired and analyzed data, and helped in the writing of the manuscript. TL performed experiments and acquired data. I-HS designed experiments and provided materials. JC designed experiments and provided feedback on the study. K-CC contributed to the conception of the study, designed experiments, interpreted the data, and wrote the manuscript.

### Conflict of Interest

The authors declare that the research was conducted in the absence of any commercial or financial relationships that could be construed as a potential conflict of interest.
