# Supplementary material for: IRF-7 Mediates Type I IFN Responses in Endotoxin-Challenged Mice
Source: Front Immunol. 2020 Apr 16;11:640. doi: 10.3389/fimmu.2020.00640 (PMC7176903; doi:10.3389/fimmu.2020.00640)
Supplement: Supplementary file 1 [file Data_Sheet_1.docx]

Supplementary Material

# Supplementary Data


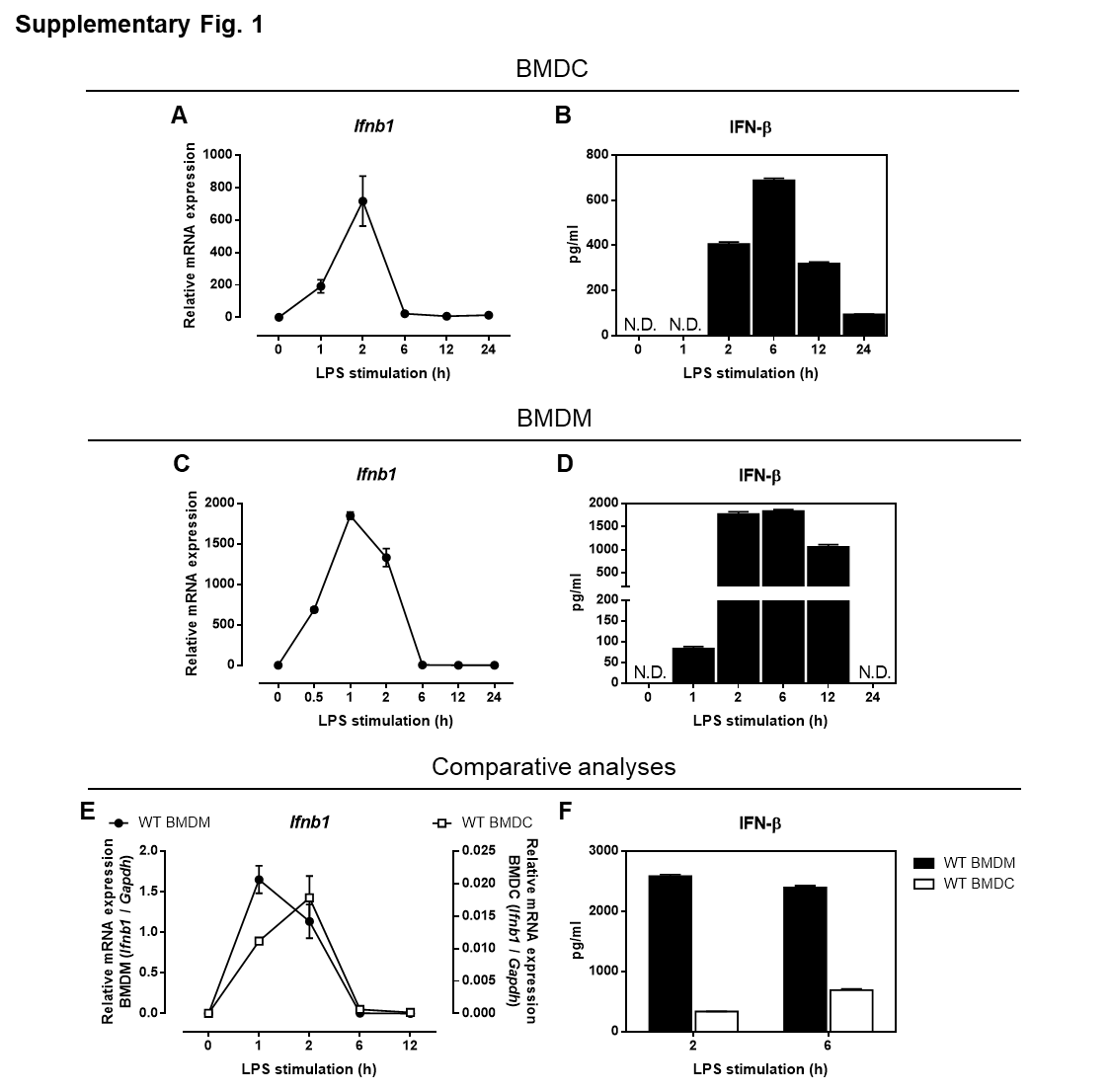


**Supplementary Figure 1.** **Magnitude and kinetics of IFN-β responses induced by LPS exposure in DCs and macrophages.** (A-B) Real-time PCR and ELISA analysis of IFN-β gene and protein expression of wild-type BMDCs, stimulated or not with 100 ng/ml LPS for 0 – 24 h. *Ifnb1* expression was normalized to *Gapdh* and expressed relative to the levels observed in un-stimulated cells. Data are presented as mean ± SD of duplicate determinations from one representative of at least three independent experiments (N.D.: not detected). (C-D) Real-time PCR and ELISA analysis of IFN-β gene and protein expression of wild-type BMDMs, stimulated or not with 100 ng/ml LPS for 0 – 24 h. *Ifnb1* expression was normalized to *Gapdh* and expressed relative to the levels observed in un-stimulated cells. Data are presented as mean ± SD of duplicate determinations from one representative of at least three independent experiments (N.D.: not detected). (E-F) Real-time PCR and ELISA analysis of IFN-β gene and protein expression of wild-type BMDMs and BMDCs, stimulated or not with 100 ng/ml LPS for 0 – 12 h. *Ifnb1* expression was normalized to *Gapdh* (left axis: 2-ΔCT in BMDMs; right axis: 2-ΔCT in BMDCs). Data are presented as mean ± SD of duplicate determinations from one representative of at least three independent experiments.


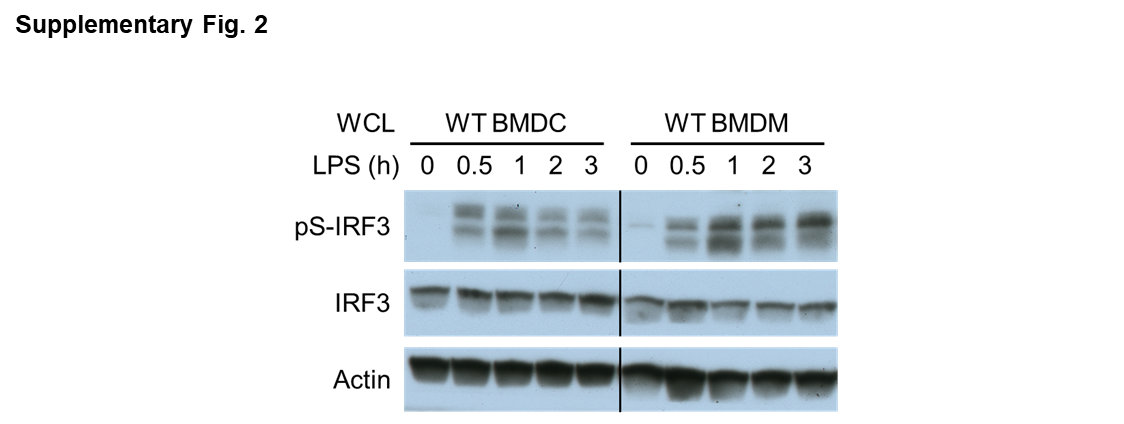


**Supplementary Figure 2.** **Comparable kinetics of IRF-3 phosphorylation in wild-type BMDMs and BMDCs.** Western immunoblot analysis of phospho-IRF3 (pS-IRF3) and total IRF3 protein expression in whole cell lysates of wild-type BMDMs and BMDCs, stimulated or not with 100 ng/ml LPS for the indicated times. Actin was used as a loading control.


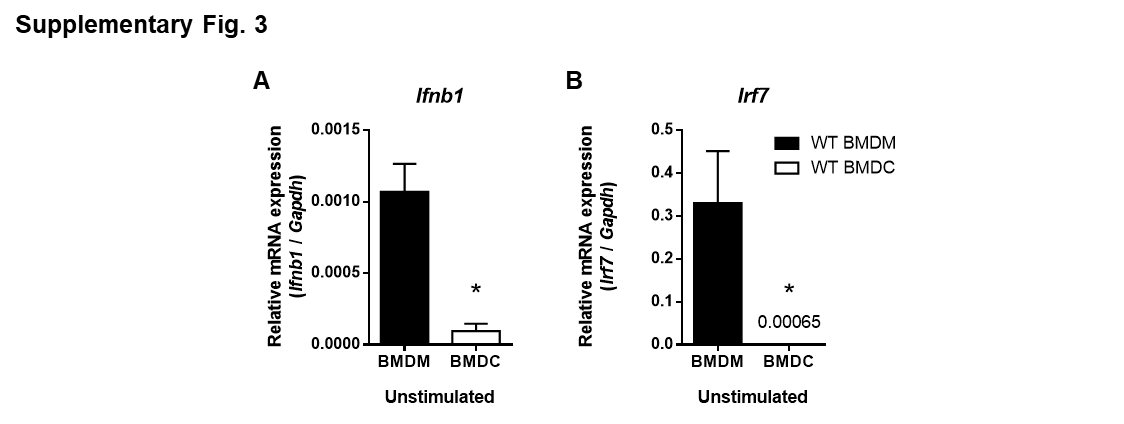


**Supplementary Figure 3.** **Resting DCs exhibit weak constitutive IFN-β production and negligible IRF-7 expression at steady-state, compared with resting macrophages.** Real-time PCR analysis of *Ifnb1* and *Irf7* gene expression in un-stimulated wild-type BMDMs and BMDCs. Gene expression was normalized to *Gapdh*. Data are presented as mean ± SEM of at least three independent experiments. Paired t-tests were used to calculate differences (p-value *<0.05).


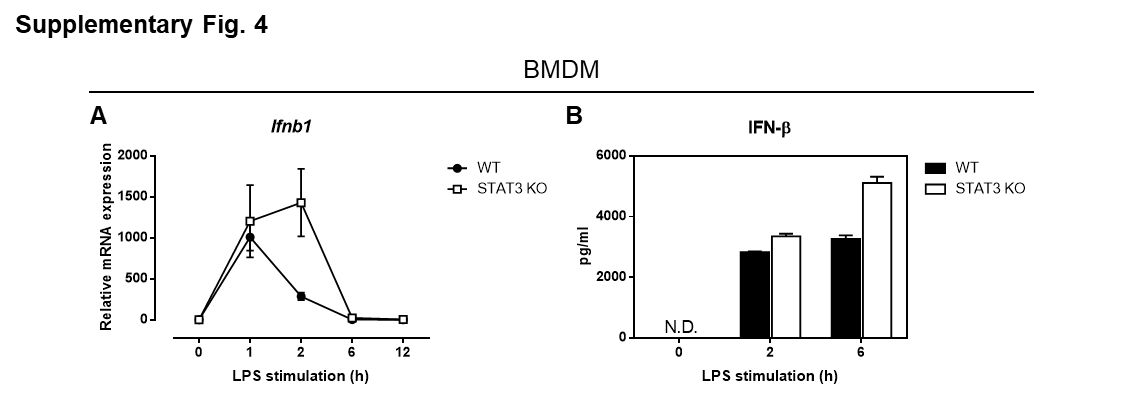


**Supplementary Figure 4.** **Autocrine/paracrine IFNAR1-STAT3 signaling is not required for IFN-β gene and protein expression in LPS-challenged macrophages.** Real-time PCR and ELISA analysis of IFN-β gene (A) and protein (B) expression of BMDMs from STAT3 knockout mice, compared to wild-type control littermates, stimulated or not with 100 ng/ml LPS for 0 – 12 h. *Ifnb1* expression was normalized to *Gapdh,* and expressed relative to the levels observed in un-stimulated wild-type control cells. Data are presented as mean ± SD of duplicate determinations from one representative of at least two independent experiments (N.D.: not detected).


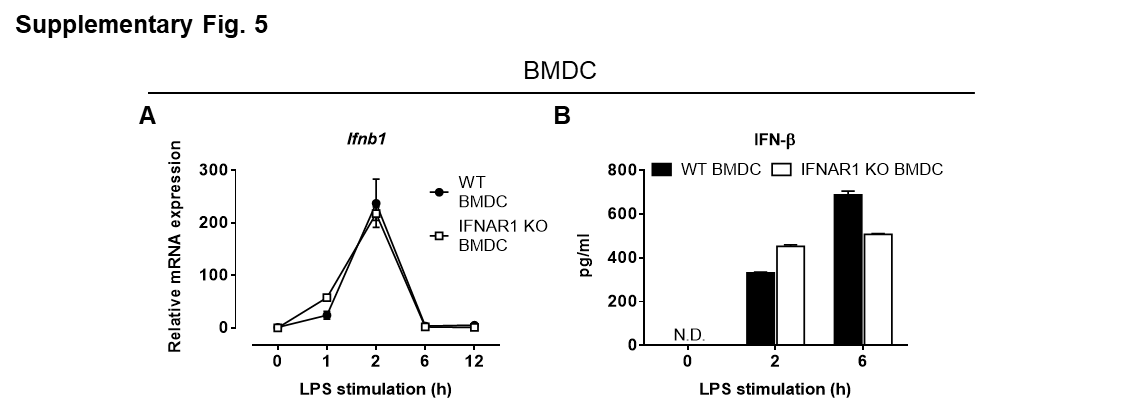


**Supplementary Figure 5.** **Autocrine/paracrine IFNAR1 signaling does not regulate IFN-β gene and protein expression in LPS-challenged DCs.** Real-time PCR and ELISA analysis of IFN-β gene (A) and protein (B) expression of BMDCs from IFNAR1 knockout mice, compared to wild-type control littermates, stimulated or not with 100 ng/ml LPS for 0 – 12 h. *Ifnb1* expression was normalized to *Gapdh* and expressed relative to the levels observed in un-stimulated wild-type control cells. Data are presented as mean ± SD of duplicate determinations from one representative of at least two independent experiments (N.D.: not detected).


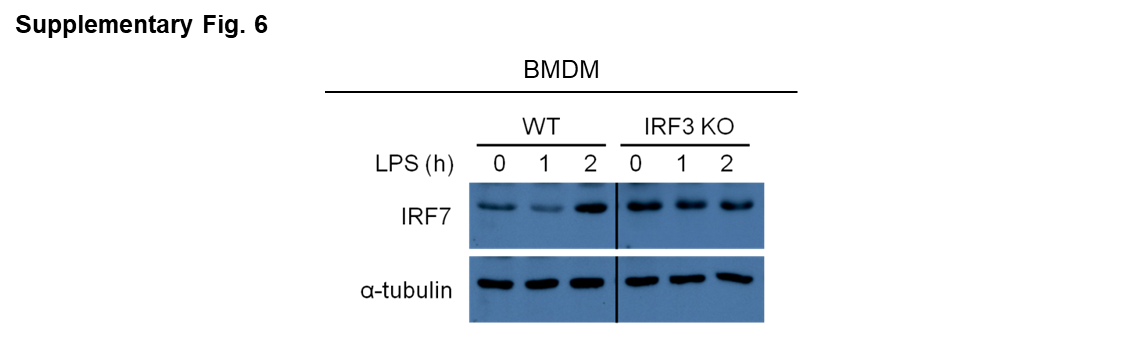


**Supplementary Figure 6.** **IRF-7 expression is essentially normal in IRF-3-null macrophages.** Western immunoblot analysis of total IRF-7 protein expression in whole cell lysates of wild-type and IRF-3 knockout BMDMs, stimulated or not with 100 ng/ml LPS for 0 – 12 h. Data are representative of at least two independent experiments.

**
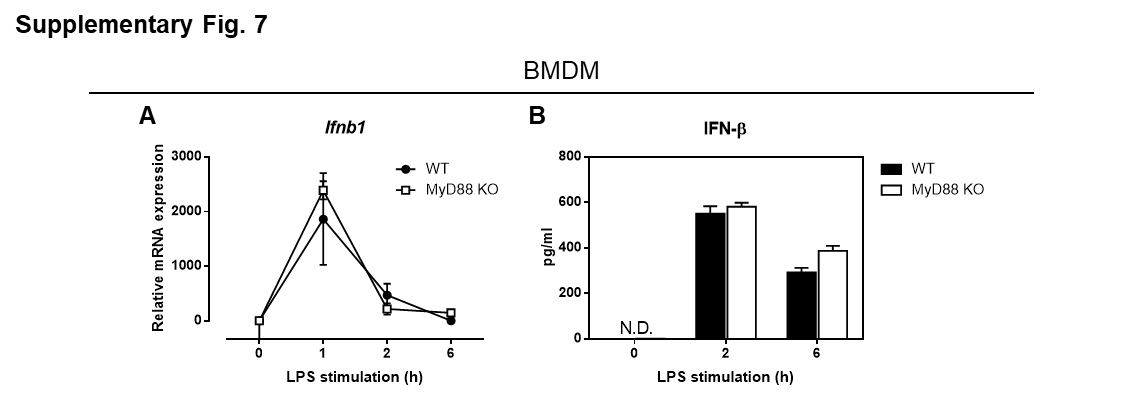
**

**Supplementary Figure 7.** **IRF-7-mediated IFN-β expression in LPS-challenged macrophages is independent of MyD88.** Real-time PCR and ELISA analysis of IFN-β gene (A) and protein (B) expression of BMDMs from MyD88 knockout mice, compared to wild-type control littermates, stimulated or not with 100 ng/ml LPS for 0 – 6 h. *Ifnb1* expression was normalized to *Gapdh* and expressed relative to the levels observed in un-stimulated wild-type control cells. Data are presented as mean ± SD of duplicate determinations from one representative of at least two independent experiments (N.D.: not detected).


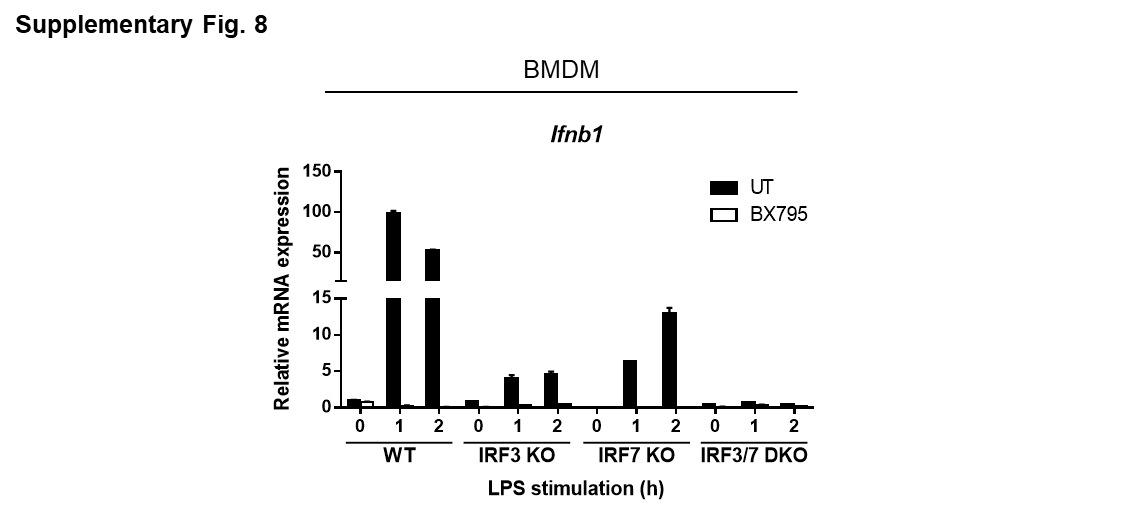


**Supplementary Figure 8.** **TBK1 is required for the activation of both IRF-7 and IRF-3 downstream of TRIF for optimal IFN-β expression in LPS-challenged macrophages.** Real-time PCR analysis of IFN-β gene expression in BMDMs from IRF-3 single knockout mice, IRF-7 single knockout mice, and IRF-3-IRF-7 double knockout mice, compared to wild-type control littermates, pre-treated or not with 2 μM BX795 (TBK1 inhibitor) for 1 h, and then stimulated or not with 100 ng/ml LPS for 0 – 2 h. *Ifnb1* expression was normalized to *Gapdh* and expressed relative to the levels observed in un-treated and un-stimulated wild-type control cells. In Fig. 2E, *Ifnb1* expression in wild-type BMDMs was induced from a CT value of about 30 in un-stimulated cells to a CT value of about 20 at 1 h post-stimulation, giving a fold-induction of 2458.7. In this figure, *Ifnb1* expression in wild-type BMDMs was induced from a CT value of about 26 in un-stimulated cells to a CT value of about 20 at 1 h post-stimulation, giving a fold-induction of 99. Hence, the discrepancy can be explained by a higher basal *Ifnb1* expression in resting BMDMs for the experiment illustrated in this figure compared with the experiment illustrated in Fig. 2E. This may be attributed to slight technical variations between different experiments, such as different batches of M-CSF-containing media, etc. The biological conclusion remains the same.

# Entire original gels


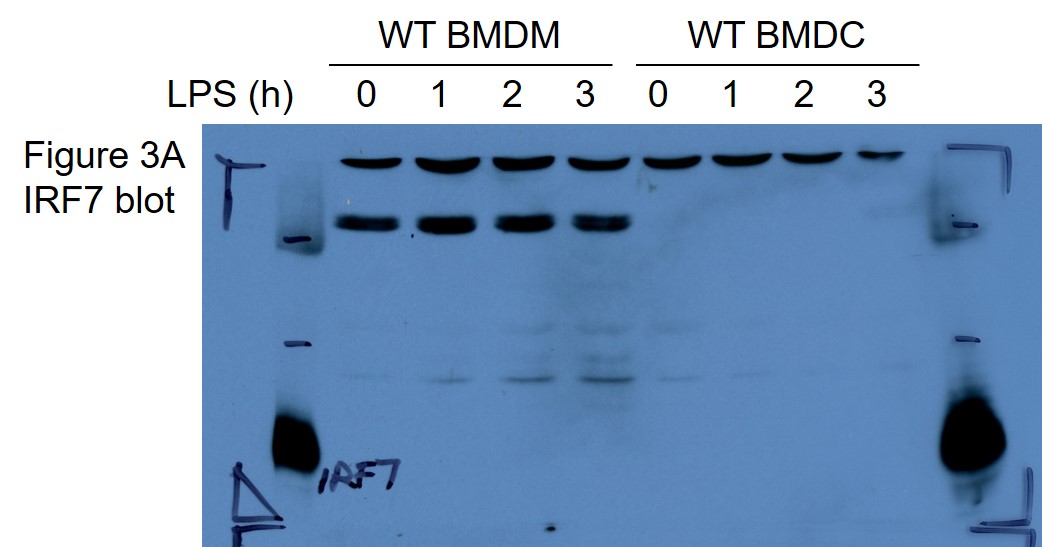


Image 1: Entire original gel of Figure 3A IRF7 blot.


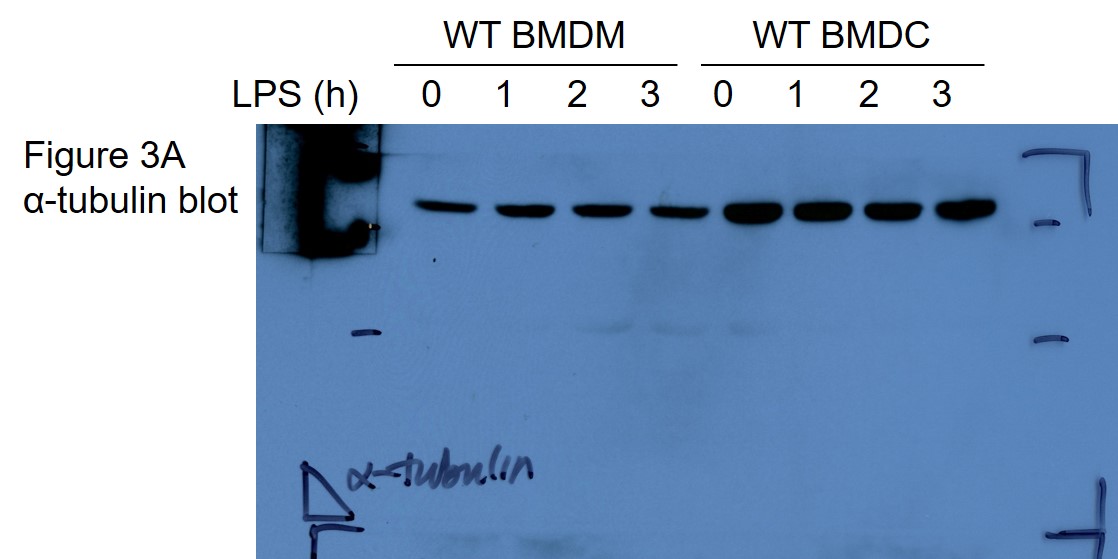


Image 2: Entire original gel of Figure 3A α-tubulin blot.


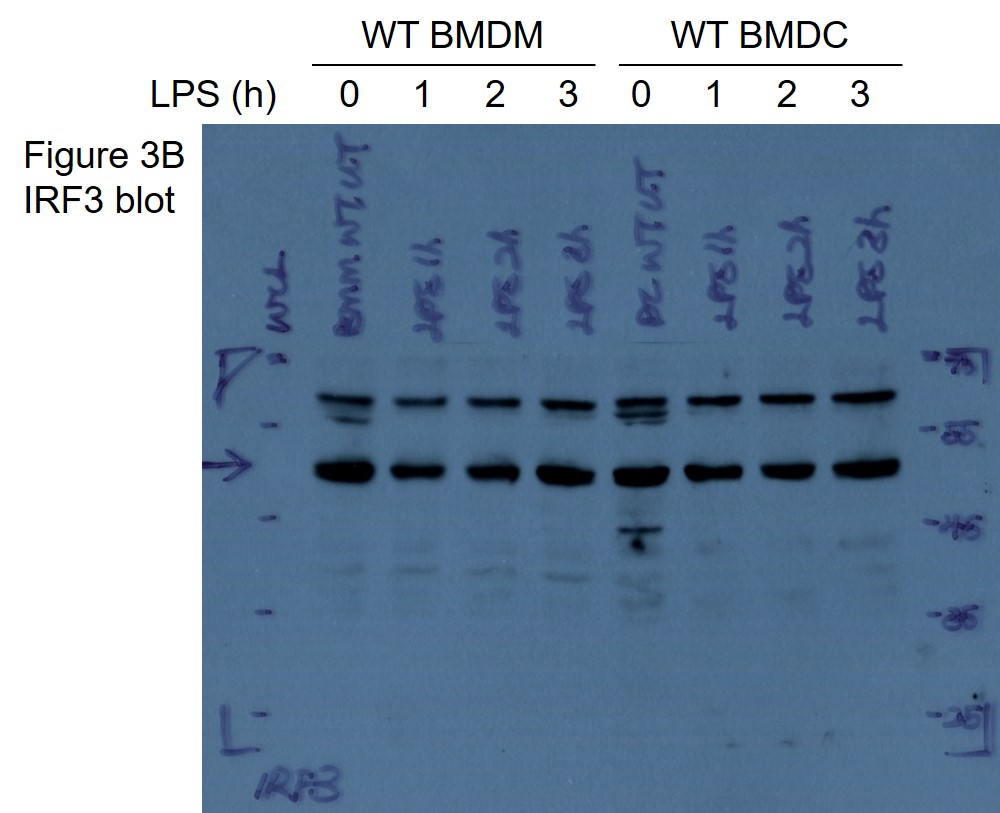


Image 3: Entire original gel of Figure 3B IRF3 blot.


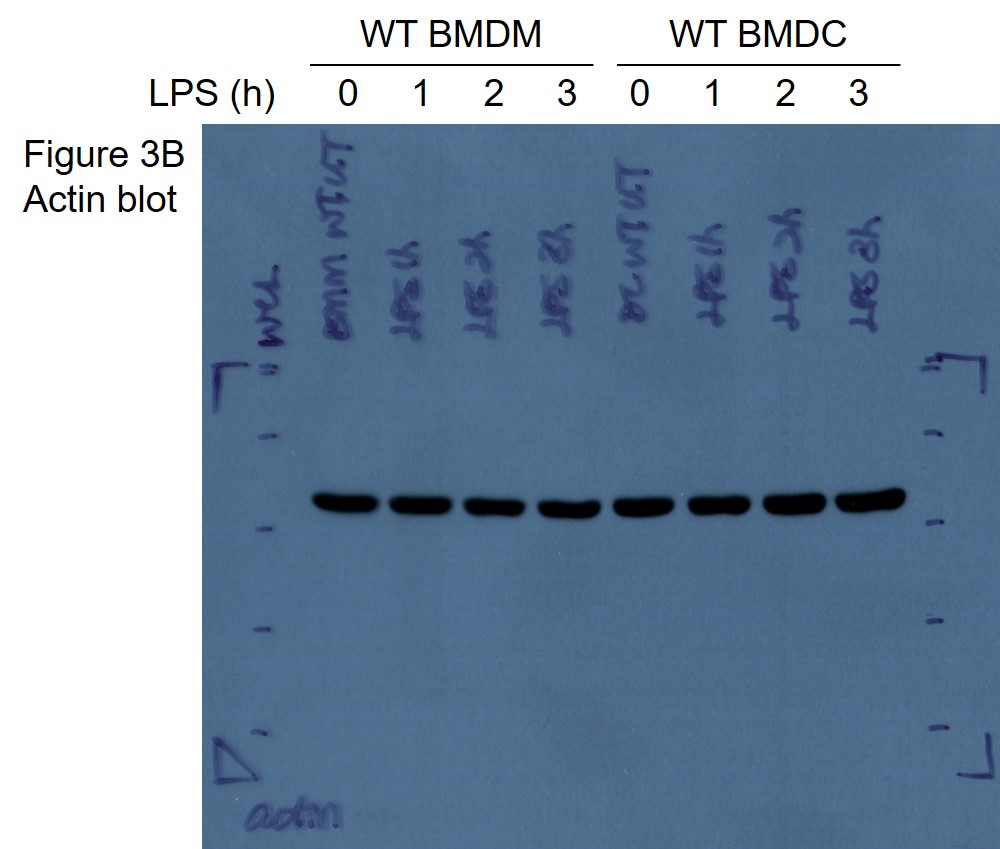


Image 4: Entire original gel of Figure 3B Actin blot.


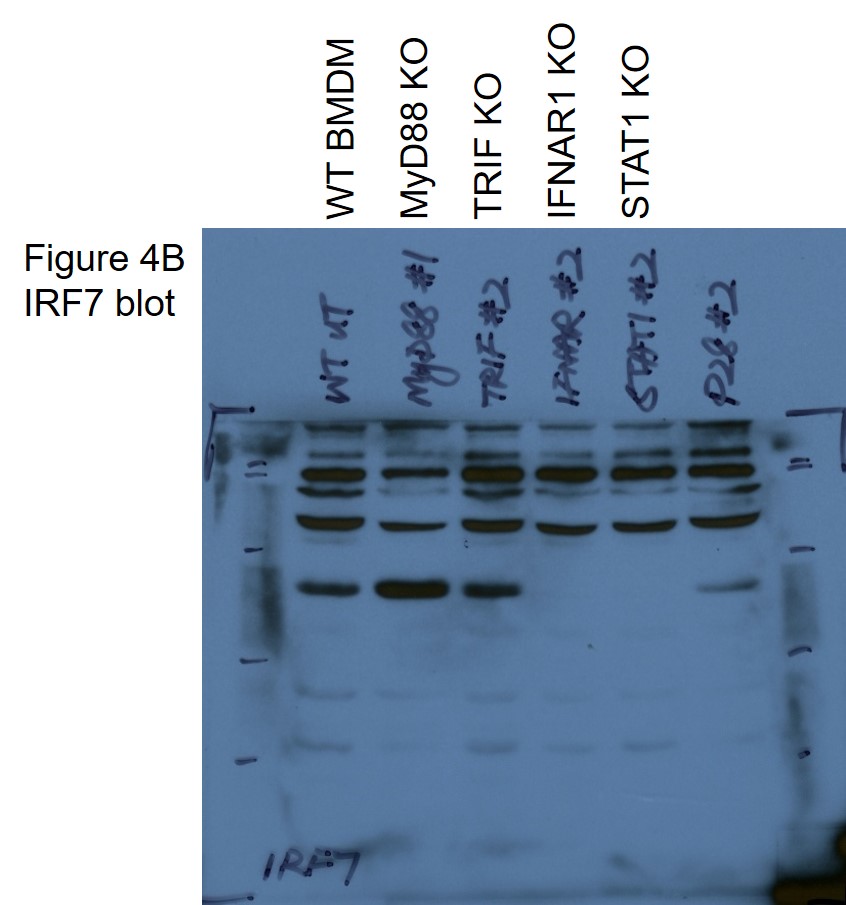


Image 5: Entire original gel of Figure 4B IRF7 blot.


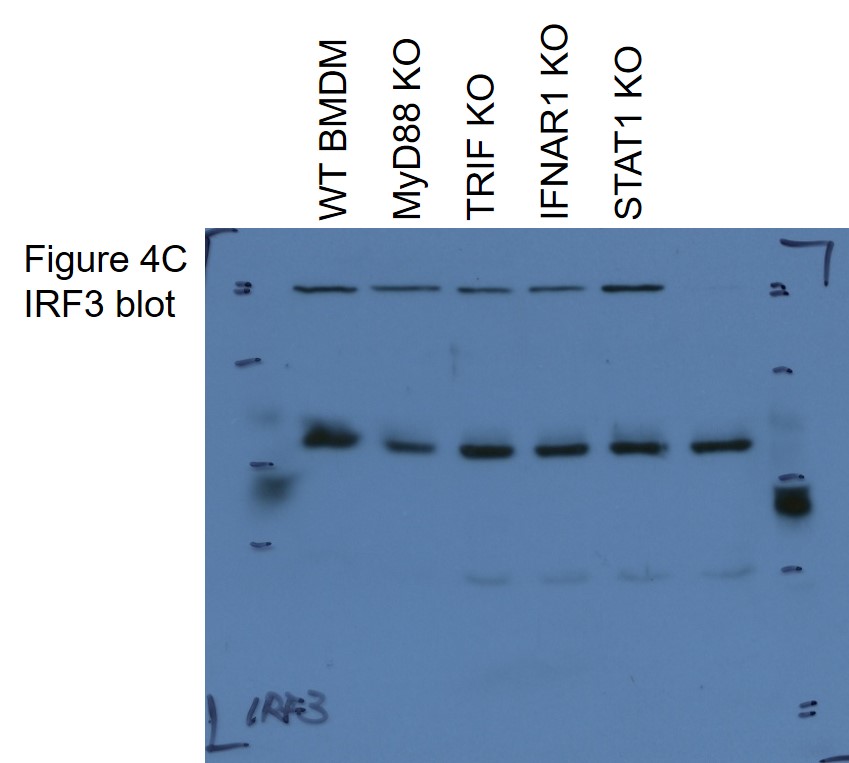


Image 6: Entire original gel of Figure 4C IRF3 blot.


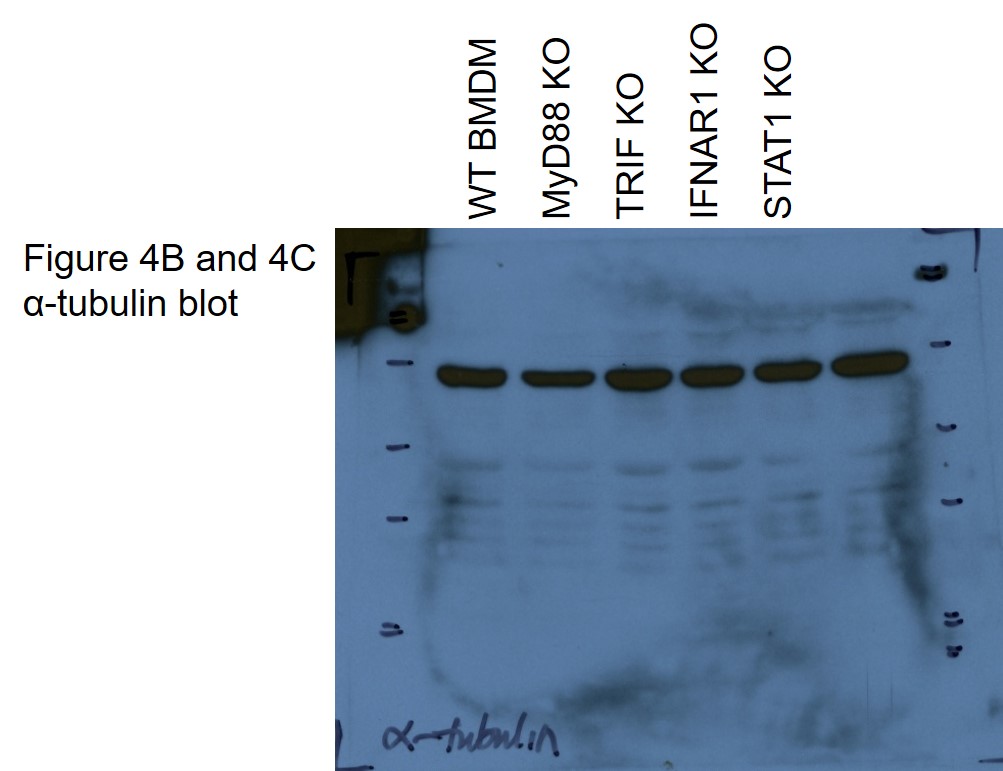


Image 7: Entire original gel of Figure 4B and 4C α-tubulin blot.


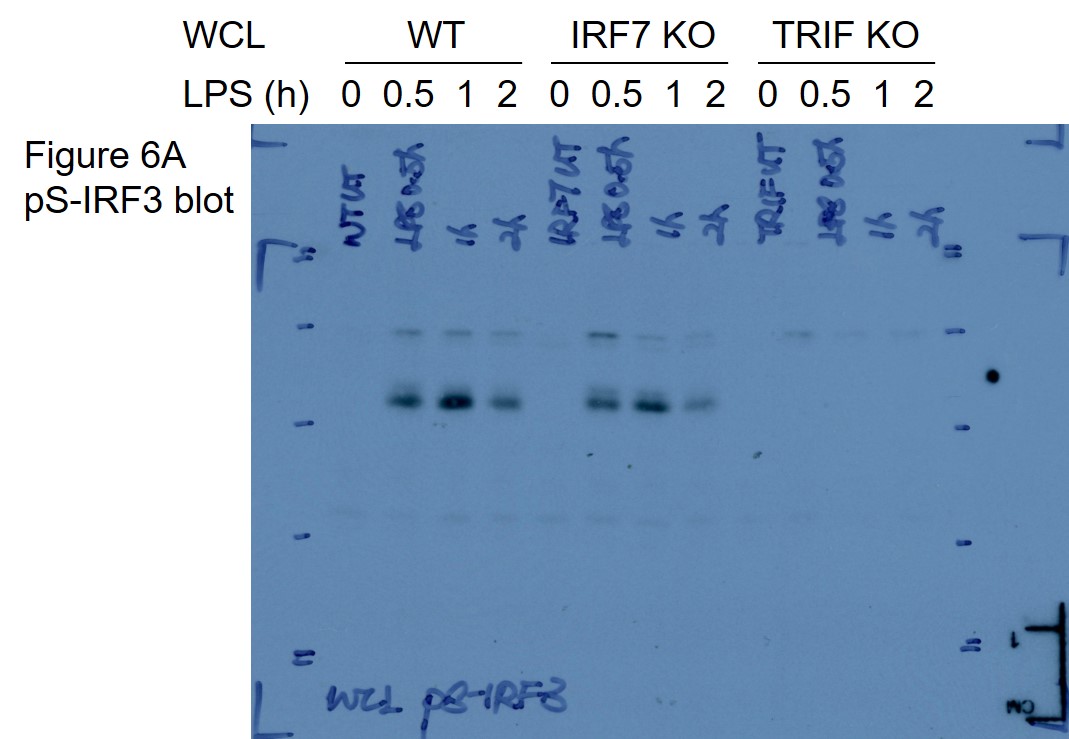


Image 8: Entire original gel of Figure 6A pS-IRF3 blot.


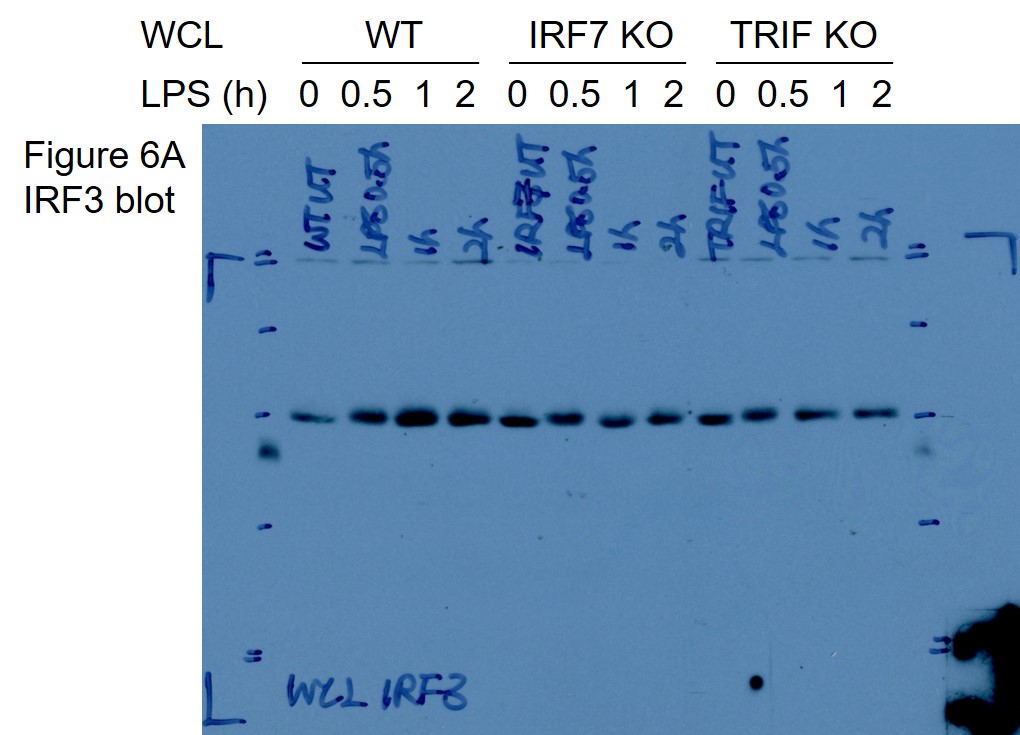


Image 9: Entire original gel of Figure 6A IRF3 blot.


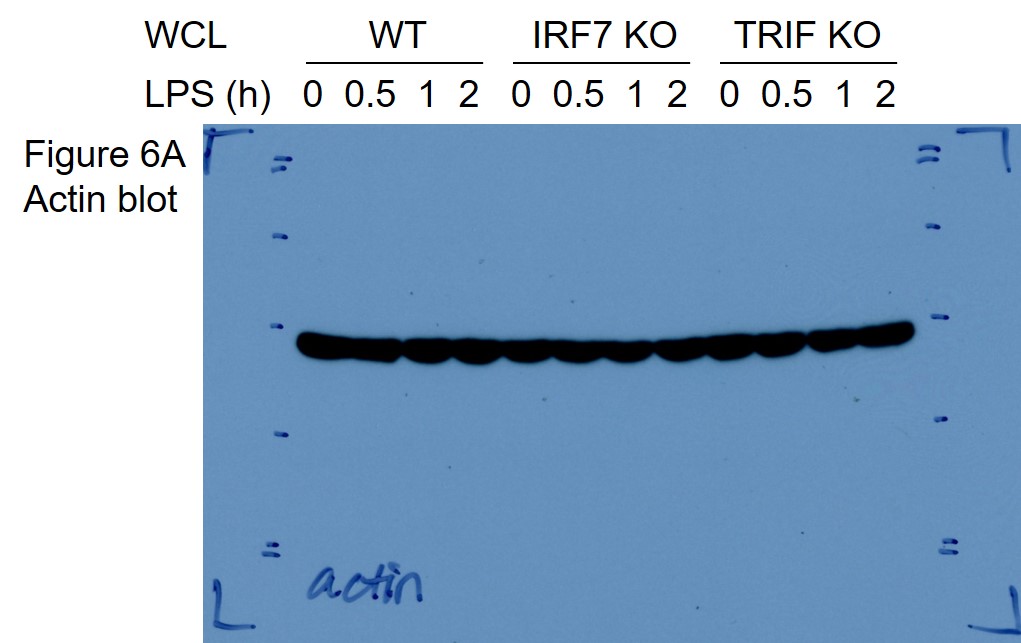


Image 10: Entire original gel of Figure 6A Actin blot.


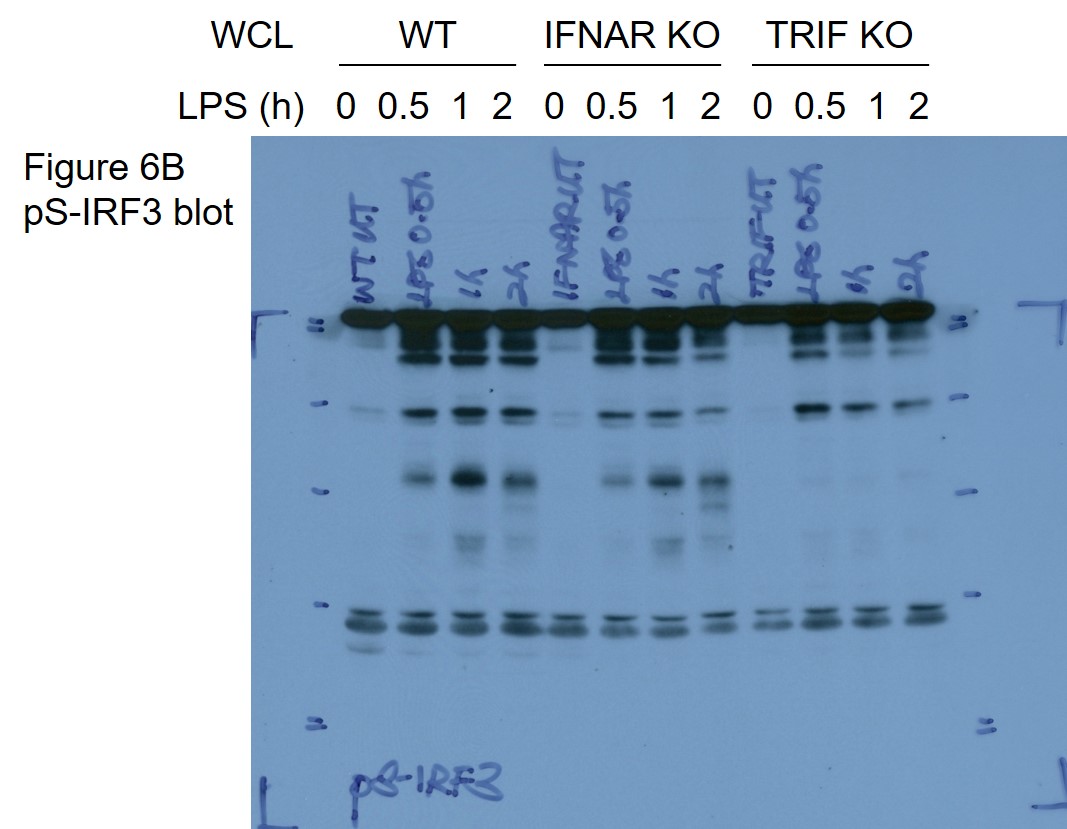


Image 11: Entire original gel of Figure 6B pS-IRF3 blot.


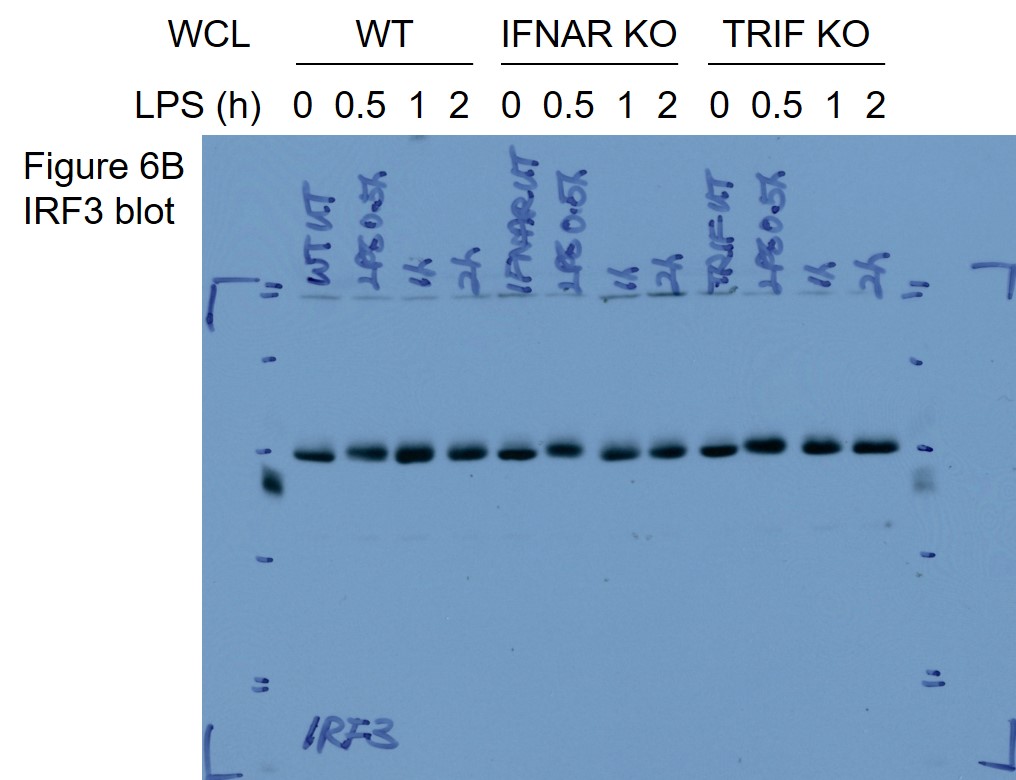


Image 12: Entire original gel of Figure 6B IRF3 blot.


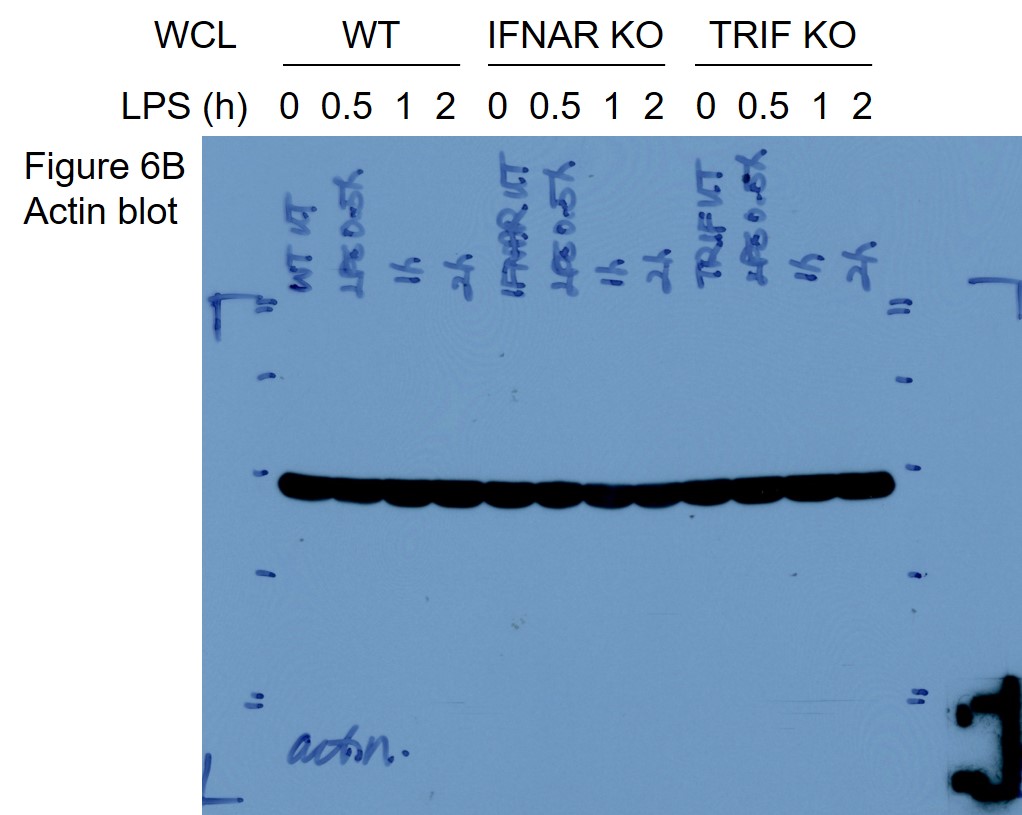


Image 13: Entire original gel of Figure 6B Actin blot.


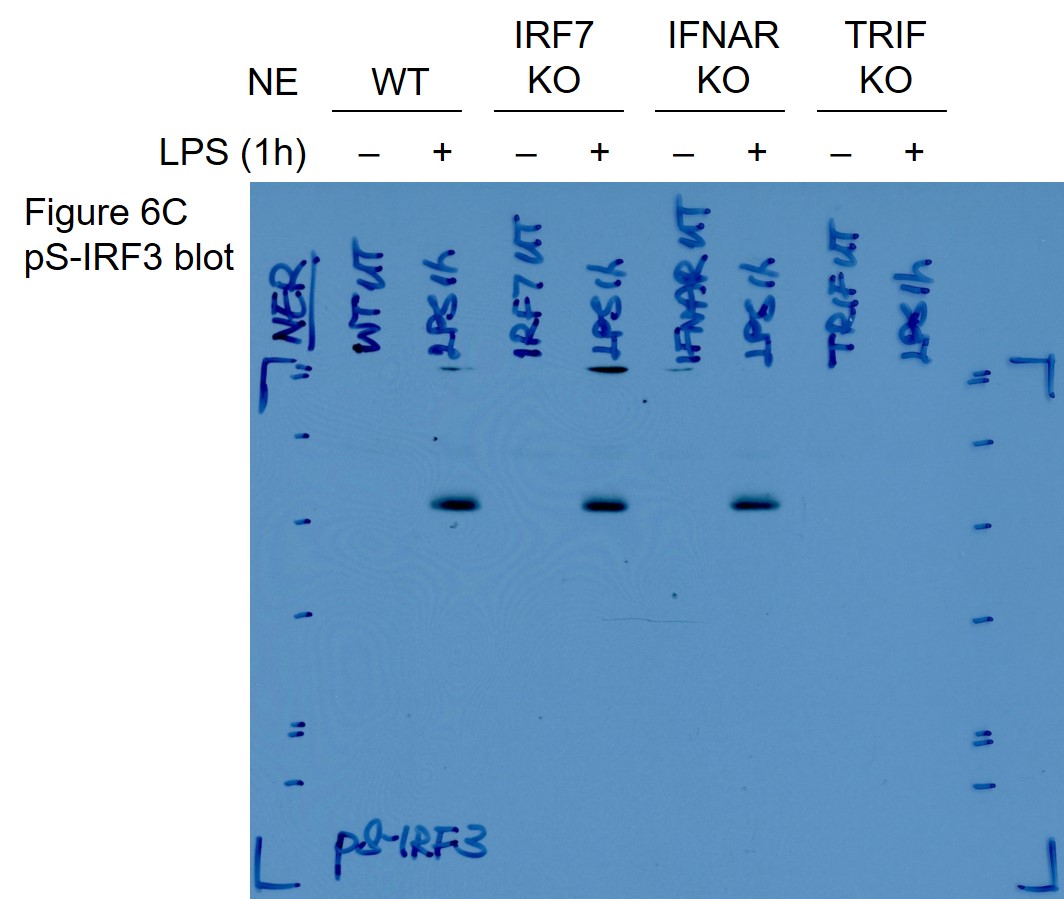


Image 14: Entire original gel of Figure 6C pS-IRF3 blot.


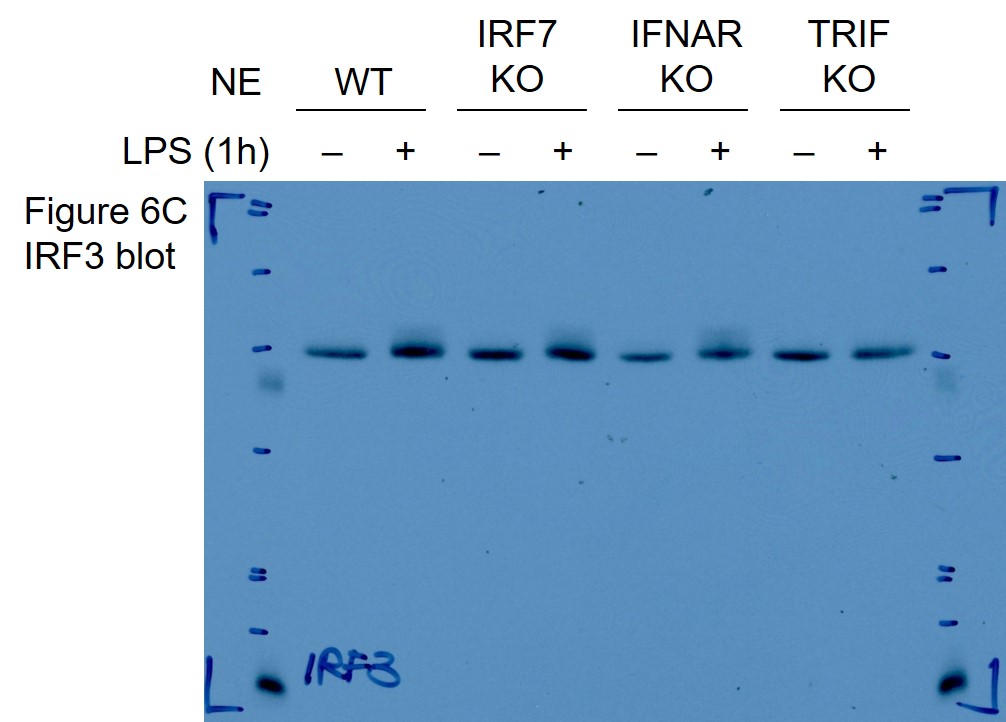


Image 15: Entire original gel of Figure 6C IRF3 blot.


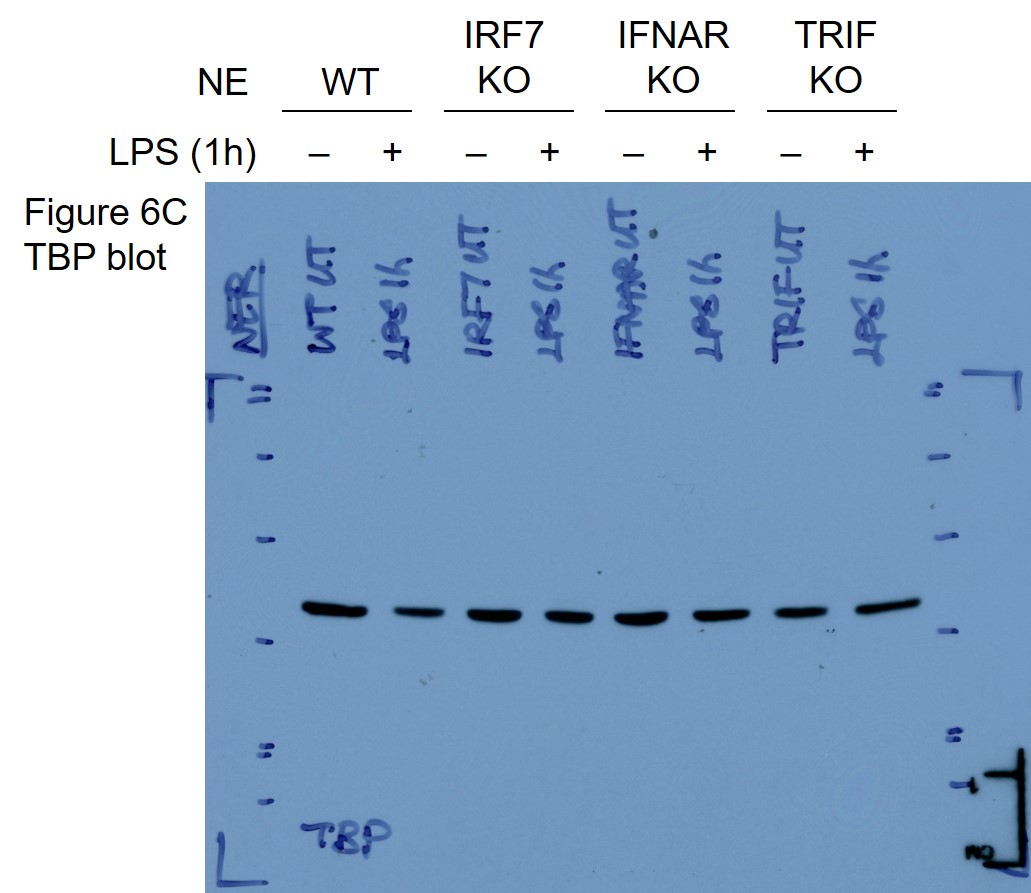


Image 16: Entire original gel of Figure 6C TBP blot.


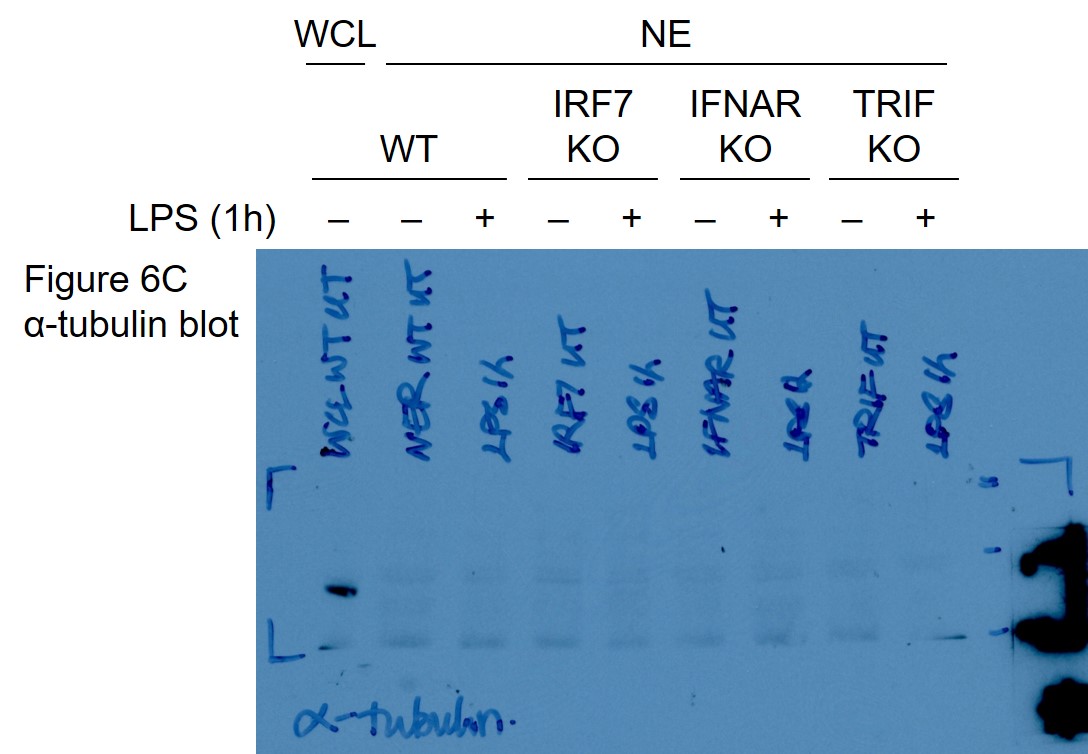


Image 17: Entire original gel of Figure 6C α-tubulin blot.


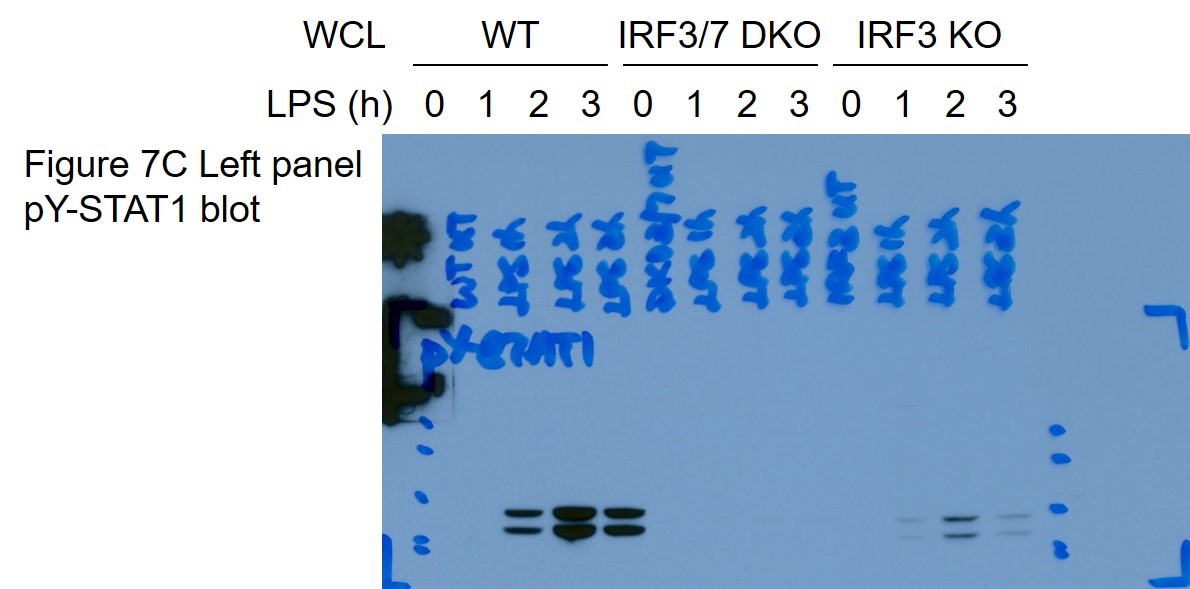


Image 18: Entire original gel of Figure 7C (left panel) pY-STAT1 blot.


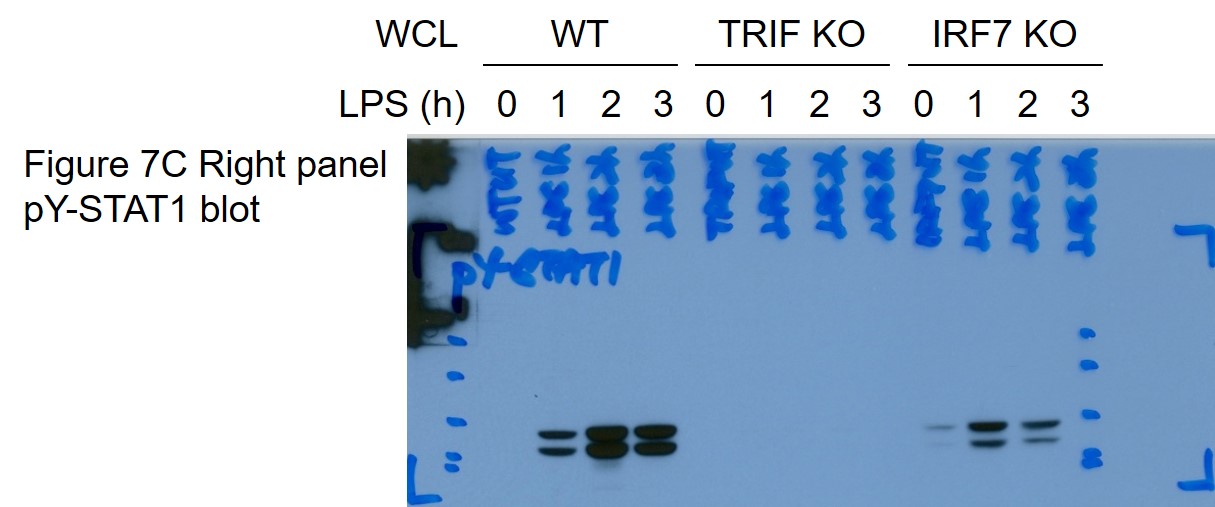


Image 19: Entire original gel of Figure 7C (right panel) pY-STAT1 blot.


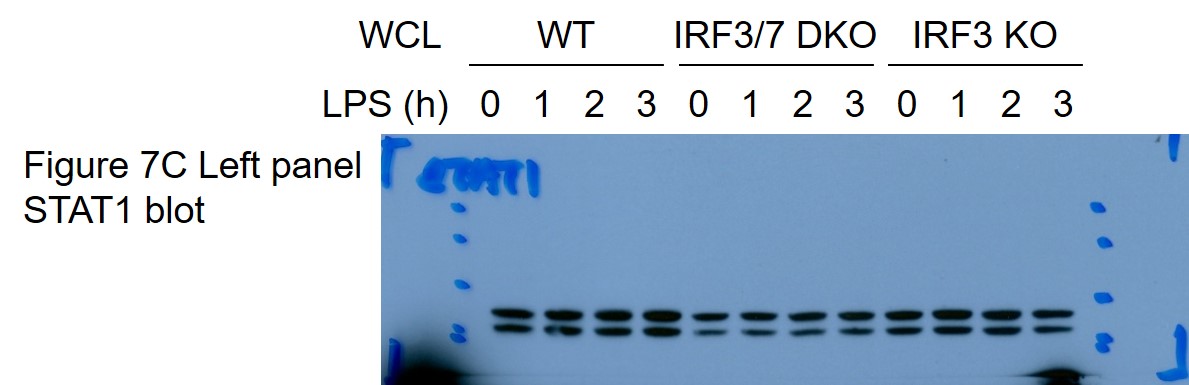


Image 20: Entire original gel of Figure 7C (left panel) STAT1 blot.


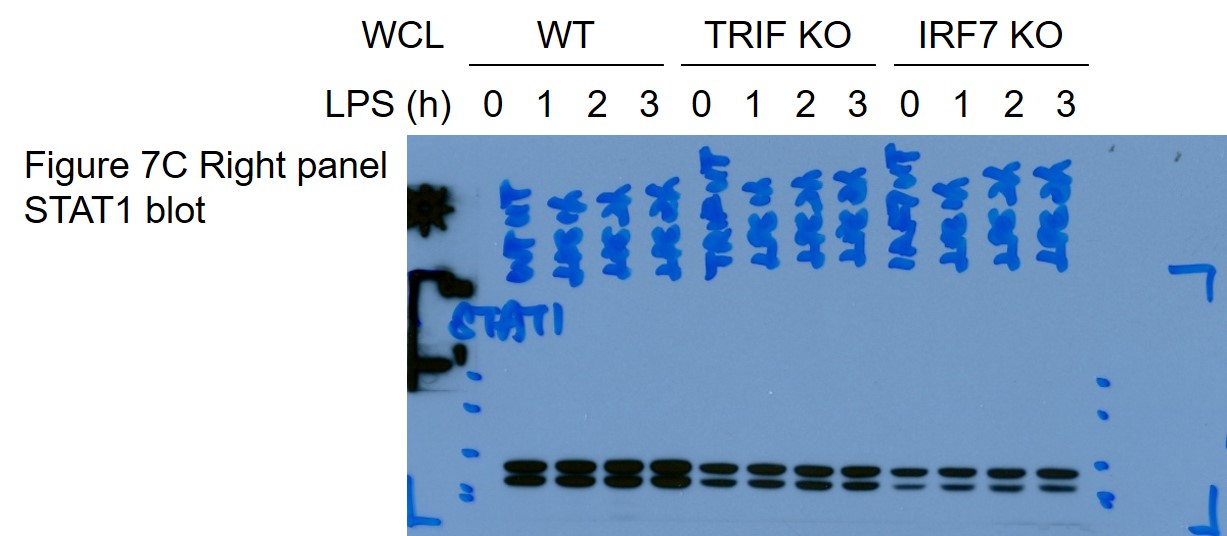


Image 21: Entire original gel of Figure 7C (right panel) STAT1 blot.


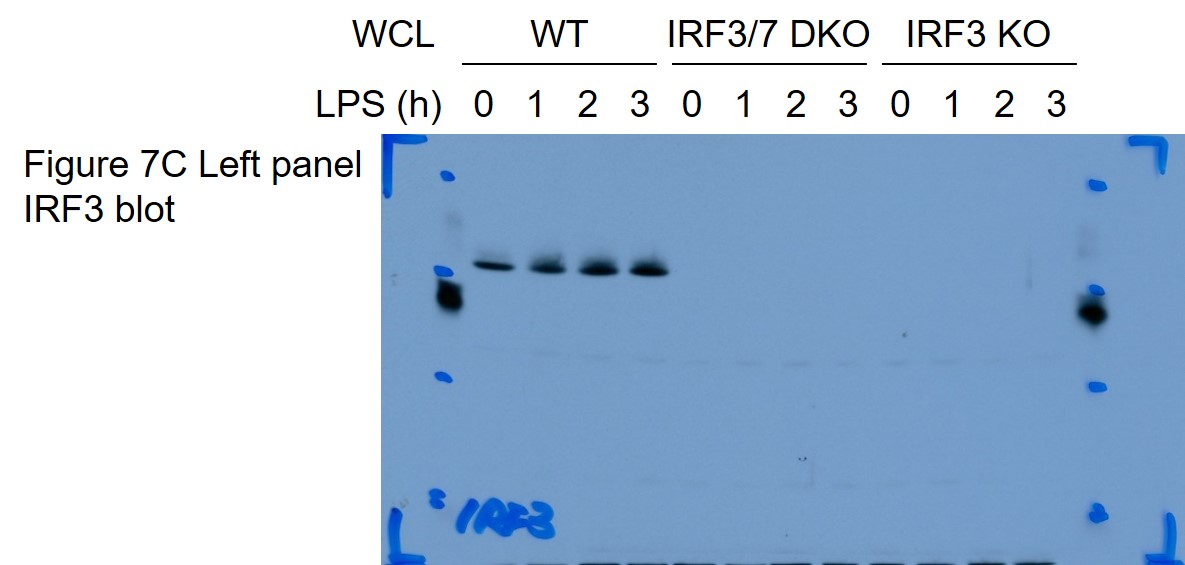


Image 22: Entire original gel of Figure 7C (left panel) IRF3 blot.


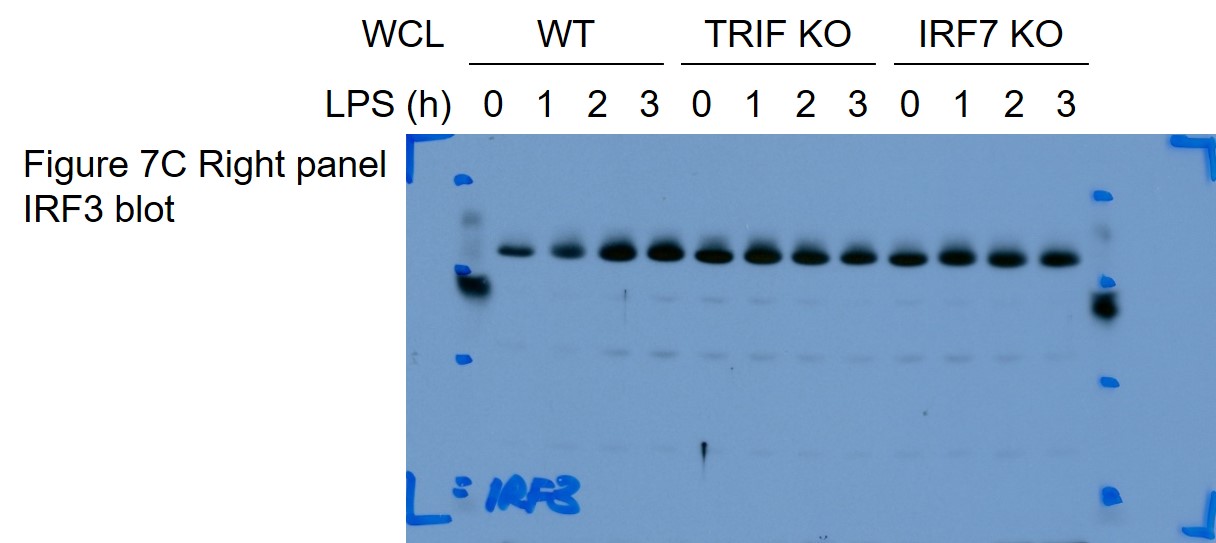


Image 23: Entire original gel of Figure 7C (right panel) IRF3 blot.


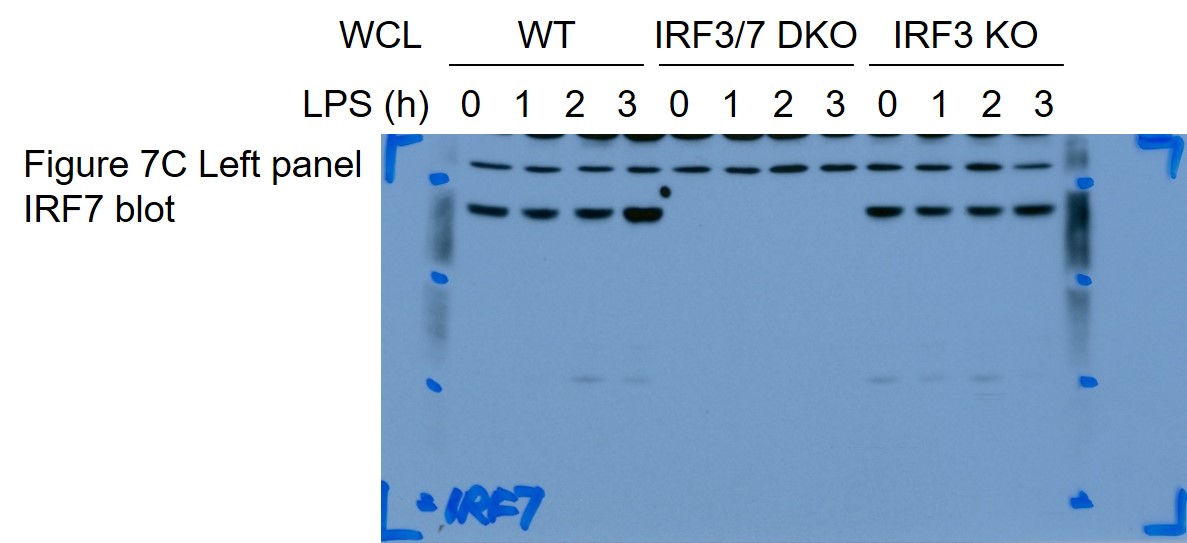


Image 24: Entire original gel of Figure 7C (left panel) IRF7 blot.


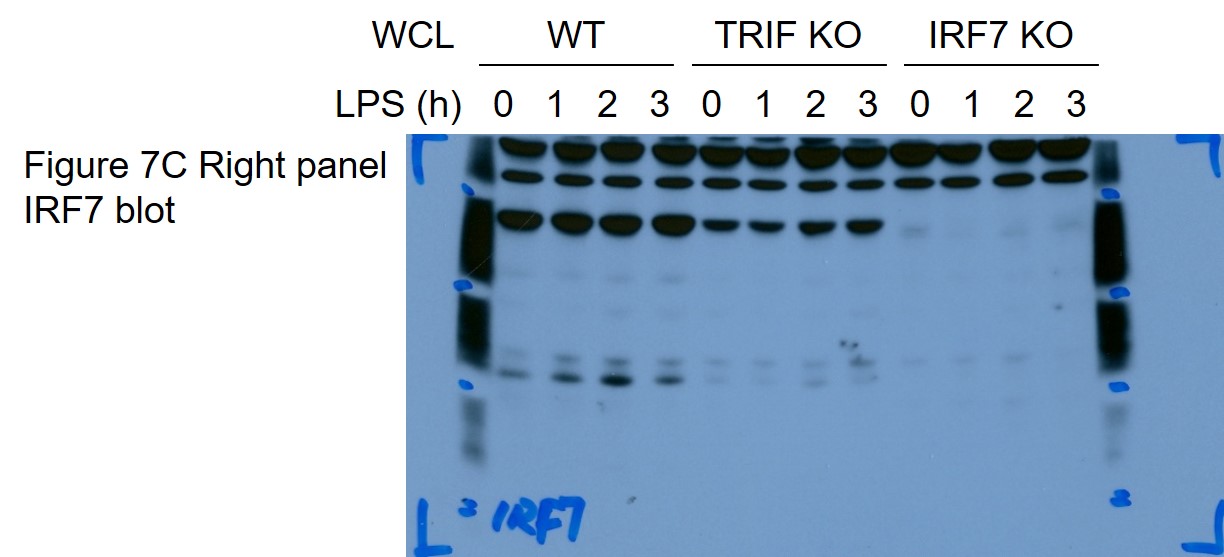


Image 25: Entire original gel of Figure 7C (right panel) IRF7 blot.


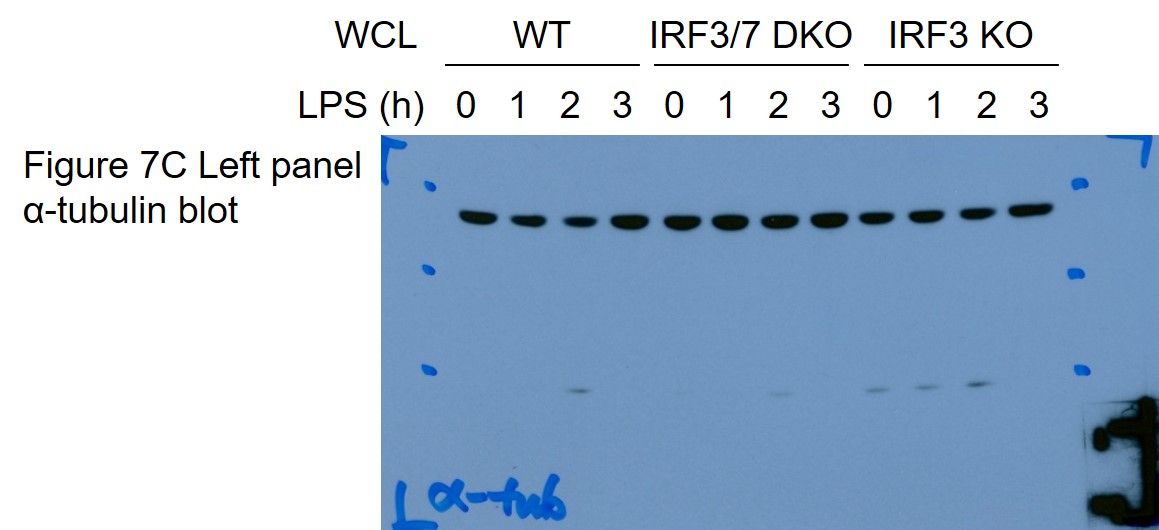


Image 26: Entire original gel of Figure 7C (left panel) α-tubulin blot.


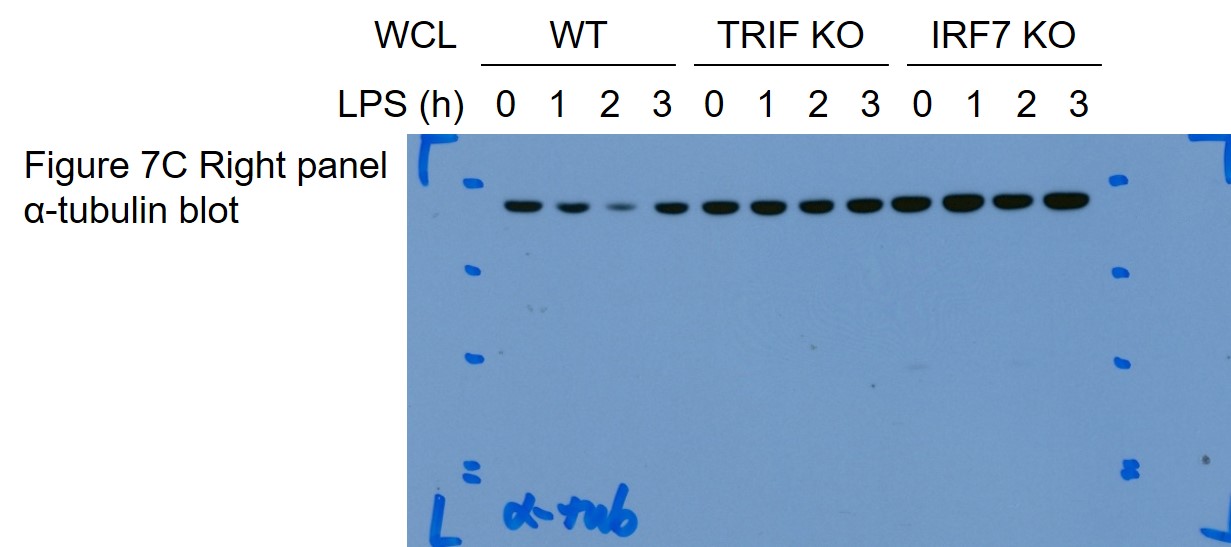


Image 27: Entire original gel of Figure 7C (right panel) α-tubulin blot.
